# Black carbon and other light-absorbing impurities in snow in the Chilean Andes

**DOI:** 10.1038/s41598-019-39312-0

**Published:** 2019-03-08

**Authors:** Penny M. Rowe, Raul R. Cordero, Stephen G. Warren, Emily Stewart, Sarah J. Doherty, Alec Pankow, Michael Schrempf, Gino Casassa, Jorge Carrasco, Jaime Pizarro, Shelley MacDonell, Alessandro Damiani, Fabrice Lambert, Roberto Rondanelli, Nicolas Huneeus, Francisco Fernandoy, Steven Neshyba

**Affiliations:** 1 Universidad de Santiago de Chile, Santiago, Chile; 2 NorthWest Research Associates, Redmond, WA USA; 3 Department of Atmospheric Sciences, University of Washington, Seattle, WA USA; 4 University of Puget Sound, Department of Chemistry, Tacoma, WA USA; 5 Joint Institute for the Study of Atmosphere and Ocean, University of Washington, Seattle, Washington USA; 6 Leibniz Universität Hannover, Institute of Meteorology and Climatology, Hannover, Germany; 7 Unidad de Glaciología y Nieves, Dirección General de Aguas (DGA), Ministerio de Obras Públicas (MOP), Santiago, Chile; 8 Centro de Investigación GAIA Antártica, Universidad de Magallanes, Punta Arenas, Chile; 9 Centro de Estudios Avanzados en Zonas Áridas (CEAZA), La Serena, Chile; 10 Center for Environmental Remote Sensing, Chiba University, Chiba, Japan; 11 Department of Physical Geography, Pontifica Universidad Catolica de Chile, Santiago, Chile; 12 Universidad de Chile, Santiago, Chile; 13 Center for Climate and Resilience Research CR2, Universidad de Chile, Santiago, Chile; 14 Laboratorio de Análisis Isotópico, Facultad de Ingeniería, Universidad Nacional Andrés Bello, Viña del Mar, Chile

## Abstract

Vertical profiles of black carbon (BC) and other light-absorbing impurities were measured in seasonal snow and permanent snowfields in the Chilean Andes during Austral winters 2015 and 2016, at 22 sites between latitudes 18°S and 41°S. The samples were analyzed for spectrally-resolved visible light absorption. For surface snow, the average mass mixing ratio of BC was 15 ng/g in northern Chile (18–33°S), 28 ng/g near Santiago (a major city near latitude 33°S, where urban pollution plays a significant role), and 13 ng/g in southern Chile (33–41°S). The regional average vertically-integrated loading of BC was 207 µg/m^2^ in the north, 780 µg/m^2^ near Santiago, and 2500 µg/m^2^ in the south, where the snow season was longer and the snow was deeper. For samples collected at locations where there had been no new snowfall for a week or more, the BC concentration in surface snow was high (~10–100 ng/g) and the sub-surface snow was comparatively clean, indicating the dominance of dry deposition of BC. Mean albedo reductions due to light-absorbing impurities were 0.0150, 0.0160, and 0.0077 for snow grain radii of 100 µm for northern Chile, the region near Santiago, and southern Chile; respective mean radiative forcings for the winter months were 2.8, 1.4, and 0.6 W/m^2^. In northern Chile, our measurements indicate that light-absorption by impurities in snow was dominated by dust rather than BC.

## Introduction

Chile is a long and narrow country spanning 38 degrees of latitude; it is therefore characterized by varied climate and varied response to climate drivers. The Andes act as a critical “water tower”, providing the main source of freshwater for Chile from snowpack and glacier runoff, wetlands and groundwater sources^1–5^ Climate warming along the Chilean Andes^6,7^ has resulted in the rapid loss of glaciers from the tropical Andes in the north^8^ to Patagonia and Tierra del Fuego in the South^9^. In central Chile, persistent drought in 2010–2015 reduced the snowpack, resulting in declines in river flow^10^. About half of the observed decline in precipitation since the 1970s in central Chile has been attributed to anthropogenic climate change^11^. Furthermore, general circulation models suggest that warming rates at higher elevations in the central Andes will outpace those for surrounding areas^2,12,13^ The past few decades have seen a regional enhancement of mid-tropospheric warming in the central Andes^13^ but the cause of the enhancement is unclear. Such warming increases the fraction of precipitation that falls as rain rather than snow, leading to earlier melting and peaks in river runoff^1^. For Chile, where the snowpack acts as a significant water reservoir, this can result in flooding in winter and spring, followed by water shortages in late spring and summer. Furthermore, model simulations predict a climatic trend of decreasing precipitation over the central and southern Andes, from 10°S to 45°S, even under strong reduction of greenhouse gas emissions^14^, with important implications for Chile’s water supply, particularly in central Chile (30–37°S) where 78% of the population is concentrated.

Because of the importance of snowpack as a source of freshwater for Chile, quantitative estimates of radiative forcing are key to predicting changes in runoff timing of snowpacks due to deposition of light-absorbing aerosol in snowpacks^15^. An important light-absorbing aerosol to quantify is black carbon (BC), an anthropogenic pollutant emitted during combustion, such as by coal-burning power plants, vehicles (particularly diesel), wood-burning stoves, agricultural fires, and forest fires^16^. Light-absorbing organic carbon (“brown carbon”) is often co-emitted with the BC as a component of carbonaceous aerosol. In the atmosphere, this carbonaceous particulate material contributes to respiratory problems and can interact with sunlight to affect climate^16^. When deposited on snow, light-absorbing carbonaceous particles reduce the albedo, leading to increased absorption of solar radiation. This triggers a positive feedback whereby the warmer snowpack ages more rapidly, further lowering albedo (through grain-size growth), ultimately accelerating surface snowmelt. Surface snowmelt then leads to the accumulation of particulate impurities at the snow surface^17^, further lowering albedo and accelerating melt. The darker underlying surface then becomes exposed earlier, leading to more absorption of sunlight and more melt^18^. Deposition of mineral dust similarly reduces snow albedo, with the same feedback processes, but scaled down by a factor of about 200 at visible wavelengths, by mass^19^. At high enough concentrations, the reduction of snow albedo by BC and mineral dust can significantly affect glacier mass balance and snow cover^18,20^. When dust thickness exceeds a critical depth of about 15 to 50 mm, it acts as an insulating layer and melting rates become lower than that of dust-free snow^21^.

It is expected that the main source of BC in northern Chile is emissions from diesel engines that power the mining industry and major astronomical observatories. Near Santiago (latitude 33.3°S), sources include emissions from transport (e.g. diesel), industrial pollution and residential heating. These emissions are trapped by the topography and the persistent wintertime temperature inversion^22^. In southern Chile (from 35°S to 47°S), the main source of heating is wood stoves, and thus the main source of particulate pollution is expected to be emissions from wood burning.

Surveys of BC in snow have been conducted in a variety of locations with various techniques, including thermo-optical methods and the SP2 instrument^16^, as well as filter-based methods, including the Light Absorption Heating Method^23^ and the Integrating Sphere Integrating SandWich (ISSW) spectrophotometer method^24–26^. The ISSW uses the wavelength dependence of measured light absorption to estimate the concentration of BC as well as the absorption Angstrom exponent (Å_tot_) of all light-absorbing insoluble particles. The latter is used to estimate the non-BC fraction of light absorption. Light-absorbing impurities in snow have been measured with the ISSW spectrophotometer in the Arctic^24,26^, northern China^27,28^ and central North America^29^. Median BC content was found to range from 3 ng/g in Greenland^26^ to 1220 ng/g in northeast China^28^.

Here, we present measurements of BC and other light-absorbing impurities made in the Chilean Andes using the ISSW spectrophotometer during two winters, one in northern Chile in 2015, and the other in central and southern Chile in 2016. As a first step toward quantifying the influence of BC in the Chilean Andes on snowmelt, we present albedo reductions and radiative forcings for a variety of snowpacks in the Chilean Andes.

The paper is organized as follows: Section 2 details the field campaigns and describes the methods of sample collection, filtration, and analysis. Sections 3–5 present results, discussion, and conclusions.

## Methods

### Sample sites

**Figure 1:**
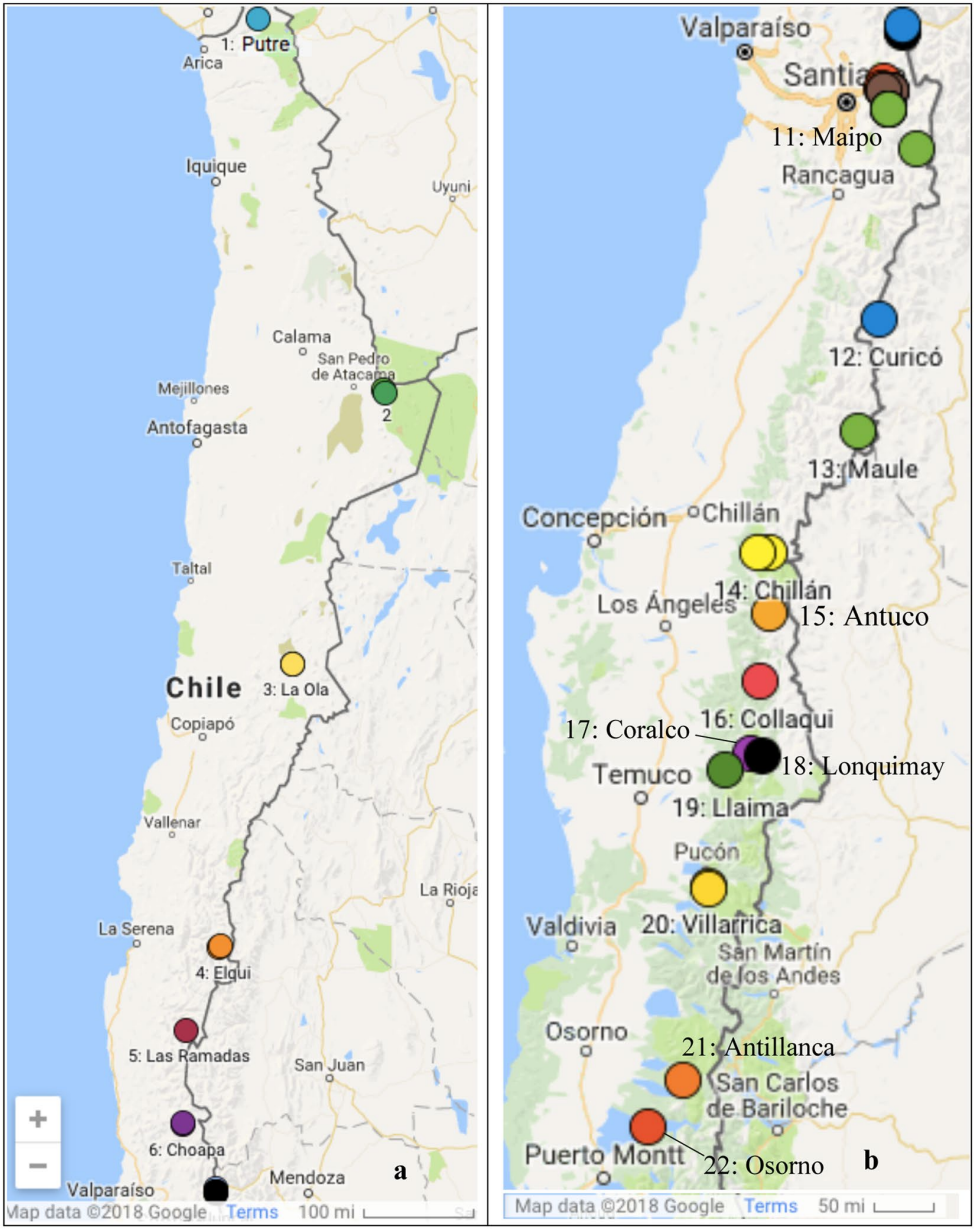
Sample sites in northern Chile (**a**) and southern Chile (**b**). Site numbers for the Santiago region are given in an expanded view in Fig. 2.

**Figure 2:**
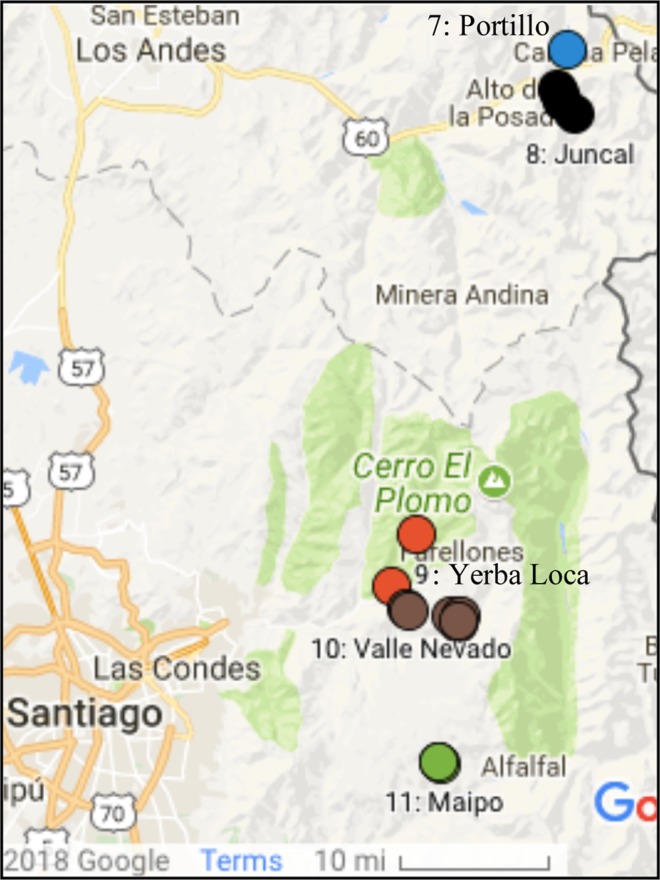
Sample sites in the region near Santiago.

**Figure 3:**
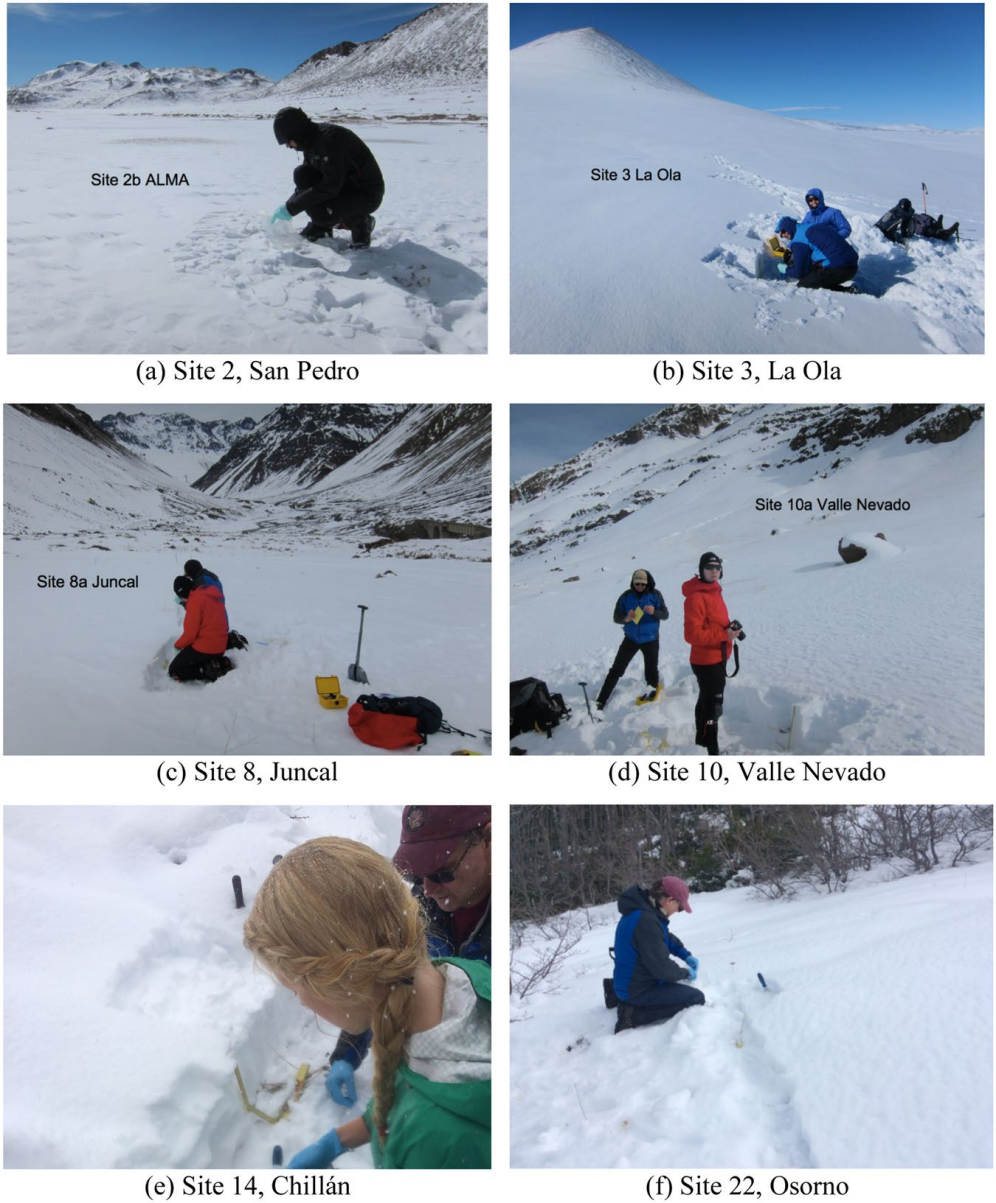
Pictures of snow sampling sites in the North (**a**,**b**), near Santiago (**c**,**d**), and in the South (**e**,**f**). Photographs were taken by the authors.

Snow was sampled at 22 locations between Putre (S 18°, W 69°) and Osorno (S 41°, W 72°). The sampling sites are assigned to one of three groups: northern Chile (18.1–32.9°S, Table 1 and Fig. 1a); the Santiago area (33.3–33.8°S, Table 2 and Fig. 2); and southern Chile (35.1–41.1°S, Table 3 and Fig. 1b). Topographic maps showing the sample sites are given in Figs S1–S2 of the Supplemental. Sampling in the north occurred in one north-south transect from 4 to 27 July 2015. Sampling in the south occurred during two north-south transects in 2016: 13–21 July and 19 August – 1 September. Sampling near Santiago was done in both winters 2015 and 2016. The individual sample sites are described below; photographs of some of the sites are shown in Fig. 3. Date of sampling, latitude, longitude, elevation and total snow depth are given for each site in Tables 1–3. Sites are listed in latitude order; for each site where sampling occurred more than once, sequential samples are listed in order of date.

**Table 1:** Snow sampling sites in northern Chile.

Site	Location	Date	Latitude (°S)	Longitude (°W)	Elevation (m)	Depth (cm)
1a	Putre	2015/07/04	18.0904	69.5368	4707	1
1b	Putre	2015/07/05	18.1024	69.5206	5318	15^*^
2a	San Pedro	2015/07/10	22.9526	67.7773	5370	80^*^
2b	San Pedro	2015/07/10	22.9989	67.7580	5062	15
3a	La Ola	2015/07/14	26.4593	69.0579	3578	13
3b	La Ola	2015/07/14	26.4593	69.0579	3578	8
3c	La Ola	2015/07/14	26.4547	69.0502	3624	35
4a	Elqui	2015/07/17	29.9452	70.078	2341	22
4b	Elqui	2015/07/17	29.9630	70.0857	2190	14
5	Las Ramadas	2015/07/19	30.9606	70.5559	1757	8
6a	Choapa	2015/07/20	32.0795	70.5927	1919	6
6b	Choapa	2015/07/20	32.0637	70.5951	1902	9

Depth indicates the total depth of the snowpack, except as noted. ^*^These two sites were on glaciers or permanent snowfields, so for them we give the sampling depth rather than the total depth.

**Table 2:** Snow sampling sites near Santiago, Chile.

Site	Location	Date	Latitude (°S)	Longitude (°W)	Elevation (m)	Depth (cm)
7	Portillo	2015/07/21	32.8337	70.1331	2800	60
8a	Juncal	2015/07/22	32.8709	70.1461	2249	28
8b	Juncal	2015/07/22	32.8744	70.1430	2285	25
8c	Juncal	2015/07/22	32.8810	70.1383	2277	20
8d	Juncal	2015/07/22	32.8937	70.1264	2322	19
9a	Yerba Loca	2016/06/18	33.2820	70.3030	2413	40
9b	Yerba Loca	2016/07/25	33.3329	70.3278	1822	10
9c	Yerba Loca	2016/07/26	33.3329	70.3278	1822	5
10a	Valle Nevado	2015/07/24	33.3545	70.3120	2435	17
10b	Valle Nevado	2015/07/24	33.3594	70.2635	2636	45
10c	Valle Nevado	2015/07/24	33.3557	70.3108	2366	32
10d	Valle Nevado	2015/07/24	33.3597	70.2528	2802	12
10e	Valle Nevado	2015/07/24	33.3661	70.2551	2554	30
10f	Valle Nevado	2016/08/18	33.3662	70.2545	2635	40
11a	Valle Maipo	2015/07/26	33.4961	70.2765	2302	30
11b	Valle Maipo^a^	2015/07/26	33.4961	70.2765	2308	15
11c	Valle Maipo	2015/07/26	33.4968	70.2743	2236	15
11d	Valle Maipo	2015/07/27	33.8074	70.0122	2392	>60

Note that locations are given in order of latitude, rather than by date. Depth indicates the total depth of the snowpack. ^a^Site 11b was about 20 m away, and about 6 m uphill, from 11a.

**Table 3:** Snow sampling sites in southern Chile.

Site	Location	Date	Latitude (°S)	Longitude (°W)	Elevation (m)	Depth (cm)
12	Curicó	2016/08/19	35.1363	70.479	1860	60
13	Maule^a^	2016/08/20	35.9888	70.5625	1860	90
14a	Chillán	2016/08/22	36.9073	71.3999	1963	40
14b	Chillán	2016/07/13	36.9216	71.4916	1554	25
15	Antuco	2016/08/23	37.3679	71.3826	1494	20
16	Collaqui	2016/08/24	37.8764	71.4741	1142	10
17	Corralco	2016/08/27	38.4059	71.5595	1601	80
18	Lonquimay	2016/08/26	38.4255	71.4604	1652	50
19	Llaima	2016/08/28	38.5272	71.7957	1830	40
20a	Villarrica	2016/07/21	39.3882	71.9618	1357	65
20b	Villarrica	2016/08/29	39.3964	71.9647	1450	90
21a	Antillanca	2016/07/19	40.7726	72.2121	1093	26
21b	Antillanca	2016/09/01	40.7861	72.1921	1349	60
22	Osorno	2016/08/31	41.1200	72.5299	1326	50

Note that locations are given in order of latitude, rather than by date. ^a^Laguna del Maule.

#### Sites in northern Chile

The Andes of northern Chile are bordered to the west by the Atacama Desert, and are characterized by being dry and dusty, with minimal vegetation. During our sampling in the far north of Chile the snow was thin and present only at higher altitude. Moving south, the snow cover and snow depth generally increased and the snow line generally dropped in altitude, allowing sampling to occur at lower altitudes, as indicated in Table 1. Sites are marked in the map in Fig. 1a. As indicated in Table 1, northern sites were visited sequentially from 4 July to 20 July 2015.

#### Sites near Santiago

Snow was sampled at five sites near Santiago, all within 1 degree of latitude of each other. Santiago is by far the largest city in Chile, with a large fraction of the country’s population (6.5 million people out of 17.9 million people in Chile as of 2017). The topography and persistent wintertime temperature inversion traps urban pollution and leads to smog, along with high levels of PM 2.5 and PM10^22^. During winter, the mixing layer height can drop to 100 m in early morning and has an average height of 200 m at local noon^30^. Within the daytime temperature inversion, the temperature increase is 3–6 K with an inversion top of 600 to 900 m^31^. Accumulations of soot particles are visible on balconies in the city. As shown in Table 2, sites near Santiago were visited in July 2015, and June, July and August 2016. Sites are marked in the map in Fig. 1b.

#### Sites in southern Chile

In contrast to northern and central Chile, southern Chile has frequent precipitation and abundant vegetation. Concentrations of mineral dust in snow are therefore expected to be low, relative to northern Chile. Southern Chile is considerably colder in winter (the average temperature in June is 17 °C in Arica and 7 °C in Osorno). Residential wood combustion is the main source of pollution for many cities in southern Chile^32^. Thus, a high incidence of black carbon is expected in snow in southern Chile, particularly relative to mineral dust. Sites are indicated on the map in Fig. 1.

### Sample Collection and Filtration

Sample collection and filtration follows the methodology of Doherty *et al*.^17,26,29^. Samples were collected at vertical intervals of 5 to 8 cm depth from the surface (exposed to the air) down to the solid ground. Typically, two parallel vertical profiles were collected, separated by a horizontal distance of 1 to 2 m. To minimize pollution due to nearby sources of transportation, sites were chosen to be approximately 1 km away from any roads when possible.

Samples were collected with a metal spatula into plastic food-handling bags, which were then packed in plastic Whirlpak bags. Food-handling bags were used for the inner bags because prior work indicated that BC adheres least to them, compared to other types of plastic^26,28^. To minimize the amount of particulates that adhered to the plastic bag with melting, the snow was stored in freezers or insulated containers, when possible, until immediately before filtration. The depth profile of snow density was also measured at each site.

Filtering was performed on a clean table or lab bench, pre-cleaned for dust and dirt. Gloves and lab coats were worn. Snow from Putre and San Pedro was filtered in temporary laboratories set up in local accommodations; snow from other locations was filtered in University laboratories. In southern Chile and near Santiago in 2016, bagged snow samples were stored in Styrofoam coolers to keep them at near-freezing temperatures during transportation to a freezer in a University; samples were thereafter kept frozen until they could be filtered in the University laboratory.

Because particulate matter adheres to glass less than it adheres to plastic, the snow was transferred to a glass beaker for melting, then the beaker was covered with a plastic bag to prevent external contamination. The snow was heated in a microwave oven until just melted, to avoid heating of the meltwater. A 0.4-µm Nuclepore filter was placed in a filter holder mounted on top of a side-arm flask. A stainless-steel funnel was clamped above the filter holder. The meltwater was then vacuum-filtered with a hand pump or electric pump through the filter. After filtering, filters were carefully placed in sterile petri dishes. The petri dishes were opened slightly to allow the filters to air dry, after which the petri dish lid was kept closed until optical analysis was performed. The mass of snow filtered was adjusted with the goal of producing filters with loadings within approximately 0.4 to 40 µg/cm^2^, to stay within the measurement-sensitivity range of the spectrophotometer (described in Section 2.3); filters determined to be outside the spectrophotometer measurement range were discarded. The meltwater volume was measured. The funnel, filter holder, and beaker were cleaned after each filtration with ultrapure water.

### Optical Analysis

Optical analysis of filters is performed with the ISSW spectrophotometer described by Doherty *et al*.^29^, which is a modified version of the instrument developed by Grenfell *et al*.^25^. The ISSW configuration includes a sample filter sandwiched between two integrating spheres, positioned above and below the sample filter. Input light is fed via fiber optics through a side port into the lower sphere, and light exits through a side port in the upper sphere to the spectrometer via a fiber optic. Baffles prevent direct light from passing through the filter and prevent light from the filter from passing directly out through the side port; thus, only diffuse light is transmitted to the detector. The ISSW has a spectral range of 450–750 nm and has a measurement resolution of 1 nm which is then averaged to 10 nm.

The spectrum of light attenuation recorded by the spectrometer needs to be calibrated in order to convert the measured signal to a loading of BC on the filter. This is done using a set of standards with a range of known loadings of fullerene, a synthetic black carbon (Alfa Aesar, Inc., Ward Hill, MA, USA), as described by Grenfell *et al*.^25^. The calibration curves express the light attenuation as a function of fullerene loading in µg/cm^2^. Assuming fullerene is a reasonable proxy for ambient black carbon, comparison of the light attenuation by a sample filter to the light attenuation by the synthetic standards yields the BC-equivalent loading of the sample filter. Calibration is done separately for each 10-nm bin, based on separate calibration curves made from the fullerene standards. This is necessary because the sample filter usually contains non-BC light-absorbing particles as well as BC particles, and the spectral shapes of non-BC and BC particles differ. Thus the BC-equivalent loading generally varies with wavelength.

Starting from the wavelength-dependent BC-equivalent loading, we calculate the quantities given in Table 4. These are described below in turn (after ref.^29^).

**Table 4:** Quantities calculated from ISSW measurements.

Quantity	Units	Description
${{\rm{C}}}_{{\rm{BC}}}^{{\rm{\max }}}$	ng/g	Maximum BC mass concentration (assuming all light absorption at 650–700 nm is due to BC).
Å_tot_	—	Absorption Angstrom exponent 450–600 nm for all insoluble particles
${{\rm{C}}}_{{\rm{BC}}}^{{\rm{est}}}$	ng/g	Estimated BC mass concentration (based on light absorption fractionated by Angstrom coefficient, assuming a value of 1 for BC and 5 for non-BC)
${{\rm{C}}}_{{\rm{BC}}}^{{\rm{equiv}}}$	ng/g	Equivalent BC mass concentration (mass of BC that would be needed to cause the 300–750 nm light absorption by all insoluble particles in the sample)
${{\rm{f}}}_{{\rm{NBC}}}^{{\rm{est}}}$	—	Estimated fraction of 300–750 nm sunlight absorption due to non-BC constituents
Integrated BC	µg/m^2^	Column-integrated BC mass in snowpack

The wavelength range given is that used to calculate the quantity, as described in the text. Mass ratios are ratios with respect to snow water mass.

${{\rm{C}}}_{{\rm{BC}}}^{{\rm{\max }}}$ is the average BC-equivalent concentration, calculated by assuming all absorption in the 650–700 nm wavelength band is due to BC. To convert from the measured fullerene-equivalent loading (µg BC-equivalent per cm^2^) to concentration, the average 650–700 nm loading is multiplied by the exposed area on the filter (cm^2^) and divided by the mass of snow water filtered (g), yielding µg/g. This is converted to ng/g, giving the mixing ratio as ng BC per g of snow water. ${{\rm{C}}}_{{\rm{BC}}}^{{\rm{\max }}}$ represents the maximum possible black carbon in the sample.

Also of interest is the amount of absorption due to non-BC particulate components. Here we use the absorption angstrom coefficient, Å_tot_, to discriminate between BC and non-BC components. Å_tot_ is defined as:

1 $${{\rm{\AA }}}_{{\rm{tot}}}=\frac{-\mathrm{ln}\,[{{\rm{\tau }}}_{{\rm{abs}}}({{\rm{\lambda }}}_{1})/{{\rm{\tau }}}_{{\rm{abs}}}({{\rm{\lambda }}}_{2})]}{\mathrm{ln}({{\rm{\lambda }}}_{1}/{{\rm{\lambda }}}_{2})}$$

Herein, Å_tot_ is calculated as a linear fit to absorption vs. wavelength in log-log space between the wavelengths λ_1_ = 450 nm and λ_2_ = 600 nm. The absorption optical depth τ_abs_ is the mass absorption coefficient of fullerene multiplied by the BC-equivalent loading. The mass absorption coefficient of fullerene is 8.9 m^2^/g at 450 nm and 6.5 m^2^/g at 600 nm. Pure BC has Å_BC_ = 1.1. For non-BC, we assume that Å_NBC_ = 5, based on prior work by Grenfell *et al*.^25^ and Doherty *et al*.^26^. The deviation of Å_tot_ from 1.1 is therefore an indication of the amount of absorption by non-BC components in the snow.

${{\rm{C}}}_{{\rm{BC}}}^{{\rm{est}}}$ is calculated following the method of Grenfell *et al*.^25^:

2 $${{\rm{C}}}_{{\rm{BC}}}^{{\rm{est}}}={{\rm{C}}}_{{\rm{BC}}}^{{\rm{\max }}}(\frac{{\langle {{\rm{\tau }}}_{{\rm{BC}}}^{{\rm{est}}}\rangle }_{650-700}}{{\langle {{\rm{\tau }}}_{{\rm{BC}}}^{{\rm{MAX}}}\rangle }_{650-700}}),$$

where

3 $${{\rm{\tau }}}_{{\rm{BC}}}^{{\rm{est}}}({{\rm{\lambda }}}_{0})={{\rm{r}}}_{{\rm{BC}}}({{\rm{\lambda }}}_{0})\times {{\rm{\tau }}}_{{\rm{tot}}}({{\rm{\lambda }}}_{0}).$$

The value r_BC_ (λ_0_) is the fraction of absorption due to BC at a given wavelength λ_0_, and it is determined by assuming that

4 $${{\rm{\AA }}}_{{\rm{tot}}}={{\rm{r}}}_{{\rm{BC}}}{{\rm{\AA }}}_{{\rm{BC}}}+(1-{{\rm{r}}}_{{\rm{BC}}}){{\rm{\AA }}}_{{\rm{NBC}}},$$

where Å_tot_ is the measured absorption Angstrom exponent of all particles on the filter and, as given above, Å_BC_ = 1.1 and Å_NBC_ = 5.0, with r_BC_ defined over the same wavelength range as the values of Å_tot_. Rearranging Eq. (4) yields r_BC_. The value for τ_tot_(λ_0_) is L_0_ β_0_, where L_0_ is the BC-equivalent loading and β_0_ is the mass absorption efficiency of fullerene at the given wavelength. Furthermore, τ as a function of wavelength is given by

5 $${{\rm{\tau }}}_{{\rm{BC}}}^{{\rm{est}}}({\rm{\lambda }})={{\rm{\tau }}}_{{\rm{BC}}}^{{\rm{est}}}({{\rm{\lambda }}}_{0})\times {({\rm{\lambda }}/{{\rm{\lambda }}}_{0})}^{-{\dot{{\rm{A}}}}_{{\rm{BC}}}}.$$

To calculate ${{\rm{\tau }}}_{{\rm{BC}}}^{{\rm{\max }}}({\rm{\lambda }})$ the first term on the right is replaced with τ_tot_(λ_0_).

${{\rm{C}}}_{{\rm{BC}}}^{{\rm{equiv}}}$ is the mass of BC that would be needed to account for the integrated 300–750 nm solar absorption by all particulates on the filter. It is calculated by weighting the spectral, all-component (total) absorption by the downwelling solar irradiance spectrum^29^. ${{\rm{C}}}_{{\rm{BC}}}^{{\rm{equiv}}}$ differs from ${{\rm{C}}}_{{\rm{BC}}}^{{\rm{\max }}}$ in that ${{\rm{C}}}_{{\rm{BC}}}^{{\rm{\max }}}$ represents the maximum mass of BC possible, whereas ${{\rm{C}}}_{{\rm{BC}}}^{{\rm{equiv}}}$ represents the amount of absorbing material present, whether BC or not, expressed as an equivalent BC mass. ${{\rm{C}}}_{{\rm{BC}}}^{{\rm{\max }}}$ is quantified at 650–700 nm, whereas ${{\rm{C}}}_{{\rm{BC}}}^{{\rm{equiv}}}$ is quantified for 300–750 nm; since non-BC components will absorb more strongly at shorter wavelengths, ${{\rm{C}}}_{{\rm{BC}}}^{{\rm{equiv}}}$ is always greater than or equal to ${{\rm{C}}}_{{\rm{BC}}}^{{\rm{\max }}}$.

The fraction of solar absorption integrated across 300–750 nm by non-BC particles, ${{\rm{f}}}_{{\rm{NBC}}}^{{\rm{est}}}$, is calculated by “(1) extrapolating the absorption spectra linearly from 450 nm down to 300 nm^25,26^; (2) weighting the non-BC absorption spectra (${{\rm{\tau }}}_{{\rm{BC}}}^{{\rm{tot}}}({\rm{\lambda }})$ − ${{\rm{\tau }}}_{{\rm{BC}}}^{{\rm{est}}}({\rm{\lambda }})$) and all-component (total) absorption spectra (τ_tot_(λ_0_)) by a downwelling solar irradiance spectrum appropriate for clear-sky, mid-latitude winter; (3) integrating these weighted spectra across the visible range; and (4) taking the ratio of the two integrals”^29^.

In addition to reporting concentrations in given snow layers, the vertically-integrated BC mass, “Int BC”, was calculated as follows: First, the snow water equivalent is calculated in each sample layer by multiplying the snow density (g/cm^3^) by the layer depth (cm). The BC amount within each sampled layer of snow is then calculated by multiplying ${{\rm{C}}}_{{\rm{BC}}}^{{\rm{est}}}$ (ng/g) by the snow water equivalent (g/cm^2^), yielding a BC mass per unit area (ng/cm^2^). This is done for each sampled layer, and the BC mass from all layers summed together to give integrated BC for the snow column, which is reported in µg/m^2^. Because it is difficult to sample all the way to the ground without contamination by soil, the BC amount for the last few centimeters is typically estimated from ${{\rm{C}}}_{{\rm{BC}}}^{{\rm{est}}}$ and the density of the layer sampled immediately above. For a given sample site, measurements within a few hundred meters, if made on the same day, are averaged. In such cases, a nearby measurement may be used to fill in missing values (e.g. in a case where filter loading from one sample was too high for an accurate measurement).

### Estimating albedo reduction and radiative forcing

**Figure 4:**
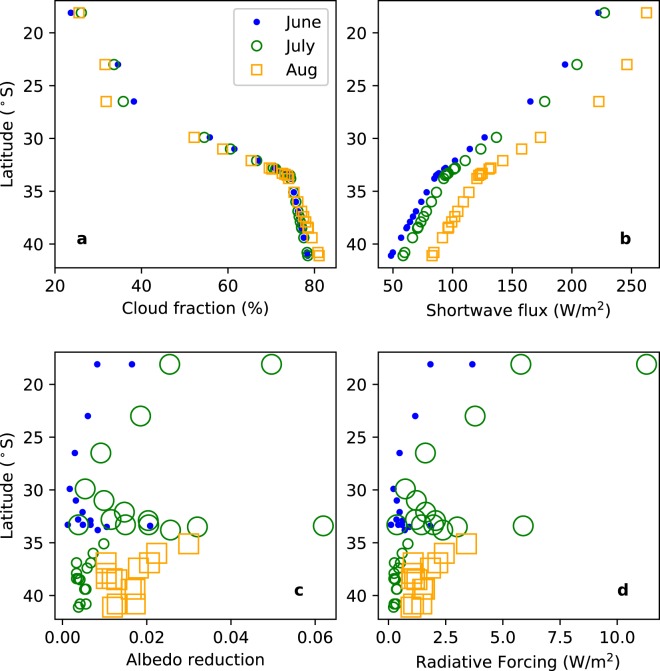
(**a**) Monthly mean cloud fraction and (**b**) Downwelling shortwave all-sky flux at the surface (CERES Terra + Aqua Edition 4 A; https://ceres.larc.nasa.gov/products-info.php?product=SYN1deg) averaged over 2000–2017. (**c**) The albedo reduction and (**d**) the radiative forcing due to light-absorbing impurities for snow-grain sizes of 100 µm (small symbols; plotted for June in the north, July in the south) and 1000 µm (large symbols; plotted for July in the north and August in the south).

The albedo reduction and radiative forcing due to light-absorbing impurities in snow were calculated using the method of Dang *et al*.^33^, and making use of the parameterization of Dang, Brandt, and Warren^19^, as follows. The all-sky broadband albedo reduction was calculated according to

6 $$\delta \alpha =CF\times \delta {\alpha }_{cloudy}+(1-CF)\delta {\alpha }_{clear},$$

where *CF* is the cloud fraction. Monthly mean cloud fraction was determined from the CERES Terra + Aqua Edition 4A^34^. Monthly means for each of the three winter months (June, July, August) were averaged from 2000 to 2017 (Fig. 4a). The albedo reduction was determined from the parameterization of Dang *et al*.^19^, which gives the albedo reduction for clear and overcast skies for a range of BC mass mixing ratios and snow grain radii. To get the albedo reduction due to BC, the albedo reduction was interpolated to the BC mass mixing ratio for a given value of ${{\rm{C}}}_{{\rm{BC}}}^{{\rm{est}}}$. This was done for cloudy skies (yielding *δα*_*cloudy*_) and for clear skies (yielding *δα*_*clear*_), after which Eq. (6) was applied. These steps were repeated for ${{\rm{C}}}_{{\rm{BC}}}^{{\rm{equiv}}}$ to calculate the albedo reduction for all light-absorbing impurities. Albedo reductions due to BC and due to all light-absorbing impurities were calculated for the months of June-July and for snow grain radii of 100 and 1000 µm.

The radiative forcing is calculated as the albedo reduction times the all-sky shortwave flux, where the latter is also taken from CERES (see above and Fig. 4b) and averaged over the years 2000 to 2017.

Albedo reductions and radiative forcings were not calculated for every site given in Tables 1–3, but rather for median values of ${{\rm{C}}}_{{\rm{BC}}}^{{\rm{est}}}$ and ${{\rm{C}}}_{{\rm{BC}}}^{{\rm{equiv}}}$ for each location (e.g. the median for all the Putre sites, etc.), except when the locations were separated by a large altitude difference or when the site was revisited on different days.

## Results

### Latitude dependence of light-absorbing impurities

**Figure 5:**
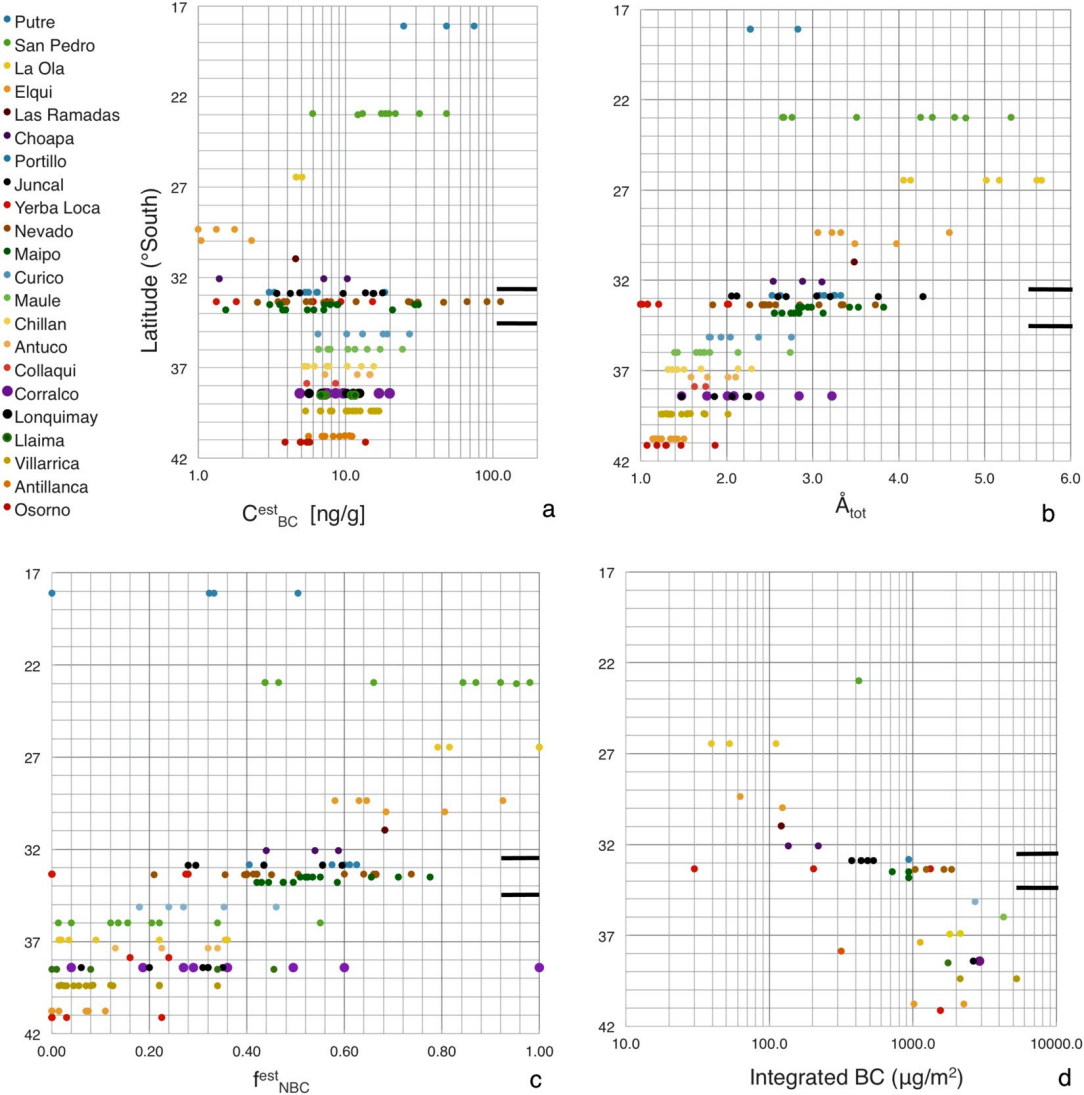
(**a**) Concentration of black carbon, (**b**) the absorption angstrom exponent, (**c**) fraction of absorption due to non-black-carbon light-absorbing impurities, and (**d**) vertically integrated black-carbon loading. Black bars to the right in each panel separate northern Chile, the Santiago region and southern Chile.

${{\rm{C}}}_{{\rm{BC}}}^{{\rm{est}}}$, Å, ${{\rm{f}}}_{{\rm{NBC}}}^{{\rm{est}}}$, and Integrated BC (Int BC) are shown as functions of latitude in Fig. 5. Sampling sites are color-coded and listed in the legend, from north to south; the color coding is the same as for the site labels in the maps shown in Figs 1 and 2. One point is plotted for each layer sampled; typically these are averages of two samples made for the same layer, although sometimes more or fewer samples were taken of a given layer. At some sites, samples were taken at different locations within the site area, or the site was revisited on a different date, as indicated in Tables 1–3.

For Fig. 5d (lower right), which shows the estimate of snow-column-integrated black carbon mass, values for sites within a few hundred meters and at a similar altitude were averaged. Multiple points are due to sample collections separated by a considerable distance, altitude, or for different conditions at the same site (e.g. La Ola, where one set of samples was made in a snow drift) or due to revisiting the site at a later date (e.g. Valle Nevado). Points are missing where snow density was not measured (e.g. Putre) or where there was no surface measurement.

### Surface snow

Table 5 gives values for Å_tot_, ${{\rm{C}}}_{{\rm{BC}}}^{{\rm{est}}}$, ${{\rm{C}}}_{{\rm{BC}}}^{{\rm{\max }}}$, and ${{\rm{C}}}_{{\rm{BC}}}^{{\rm{equiv}}}$ for surface samples at each location. The layer thickness of the surface sample is also shown in the table. Medians were calculated across all surface samples from a given location, except when the locations were separated by a large altitude difference or when the site was revisited on different days. (These values are given for each layer in each sampling site in Tables S1–S3 of the Supplemental).

**Table 5:** Median values at each location for the surface layer of snow.

Dates	Location	Latitude (°S)	Elevation (m)	Sample Depth (cm)	Å_tot_	${{\bf{C}}}_{{\bf{BC}}}^{{\bf{est}}}$ (ng/g)	${{\bf{C}}}_{{\bf{BC}}}^{{\bf{\max }}}$ (ng/g)	${{\bf{C}}}_{{\bf{BC}}}^{{\bf{equiv}}}$ (ng/g)
2015/07/04	Putre	18.1	4700	0–5	2.8	25	34	50
2015/07/05	Putre	18.1	5300	0–5	2.3	74	93	121
2015/07/10	San Pedro	23.0	5200	0–5	4.5	15	81	166
2015/07/14	La Ola	26.5	3600	0–7	4.1	5	12	23
2015/07/17	Elqui	29.9	2300	0–5	4.0	2	5	10
2015/07/19	Las Ramadas	31.0	1800	0–4	3.5	5	8	15
2015/07/20	Choapa	32.1	1900	0–5	2.8	9	13	19
2015/07/21	Portillo	32.8	2800	0–5	2.6	6	8	11
2015/07/22	Juncal	32.9	2300	0–5	2.4	15	21	25
2016/06/18	Yerba Loca	33.3	2400	0–8	2.0	15	17	21
2016/07/25	Yerba Loca	33.3	1800	0–5	1.0	1	1	1
2016/07/26	Yerba Loca	33.3	1800	0–5	2.0	9	10	13
2015/07/24	Nevado	33.4	2500	0–10	3.3	91	153	279
2015/07/26	Maipo	33.5	2300	0–5	3.7	30	72	121
2015/07/27	Maipo	33.8	2400	0–5	3.1	21	32	50
2016/08/19	Curicó	35.1	1860	0–10	2.8	27	31	50
2016/08/20	Maule	36.0	1860	0–10	2.1	17	19	26
2016/07/13	Chillán	36.9	1600	0–5	1.5	5	6	6
2016/08/22	Chillán	36.9	2000	0–10	1.7	15	16	20
2016/08/23	Antuco	37.4	1500	0–5	1.8	12	13	15
2016/08/24	Collaqui	37.9	1100	0–5	1.6	5	6	6
2016/08/27	Corralco	38.4	1600	0–10	1.5	5	5	5
2016/08/26	Lonquimay	38.4	1700	0–5	1.5	6	6	6
2016/08/28	Llaima	38.5	1800	0–5	2.1	7	9	12
2016/07/21	Villarrica	39.4	1400	0–5	1.7	10	10	12
2016/08/29	Villarrica	39.4	1500	0–5	2.0	11	13	17
2016/07/19	Antillanca	40.8	1100	0–5	1.5	11	12	12
2016/09/01	Antillanca	40.8	1300	0–5	1.1	7	7	7
2016/08/31	Osorno	41.1	1300	0–5	1.2	5	5	5

### Regional results

Regional averages and standard deviations for measurements in northern Chile, the Santiago region, and southern Chile are given in Table 6. In northern and southern Chile, where there were sometimes large distances between sites, the average and standard deviation for each site were geographically weighted by the increment of latitude that the site was judged to represent. Values shown in the table include surface measurements of Å_tot_, ${{\rm{C}}}_{{\rm{BC}}}^{{\rm{est}}}$, ${{\rm{C}}}_{{\rm{BC}}}^{{\rm{\max }}}$, and ${{\rm{C}}}_{{\rm{BC}}}^{{\rm{equiv}}}$(as well as the subsurface values for ${{\rm{C}}}_{{\rm{BC}}}^{{\rm{est}}})$, the total snow depth, snow water equivalent, integrated black carbon, and average black carbon. For subsurface and snowpack measurements, averages in the north exclude Putre, where snow density was not measured. Also shown in Table 6 are the albedo reductions due to light-absorbing impurities in snow for BC alone and for all light-absorbing impurities and the resulting radiative forcing; these are discussed below.

**Table 6:** Regional averages and standard deviations.

	Northern Chile	Santiago Region	Southern Chile
Latitude (°S)	18.1–32.1	32.8–33.5	35.1–41.1
Å_tot_	3.8 ± 0.2	2.6 ± 0.7	1.9 ± 0.2
${{\rm{C}}}_{{\rm{BC}}}^{{\rm{est}}}$ (ng/g)	15 ± 4	28 ± 35	13 ± 3
${{\rm{C}}}_{{\rm{BC}}}^{{\rm{\max }}}$ (ng/g)	40 ± 9	45 ± 61	15 ± 3
${{\rm{C}}}_{{\rm{BC}}}^{{\rm{equiv}}}$ (ng/g)	72 ± 17	75 ± 115	20 ± 6
${{\rm{C}}}_{{\rm{BC}}}^{{\rm{est}}}$, subsurf (ng/g)	15 ± 5	7 ± 3	11 ± 1
Depth^a^ (cm)	12 ± 1	38 ± 21	54 ± 9
SWE^b^ (kg/m^2^)	100 ± 21	98 ± 71	330 ± 110
Int BC^c^ (µg/m^2^)	207 ± 46	780 ± 510	2500 ± 440
Ave BC^d^ (ng/g)	6 ± 1	11 ± 11	12 ± 1
*δα*_*BC*_,^e^ 100 µm (10^−4^)	54	92	60
*δα*_*BC*_, 1000 µm (10^−4^)	170	280	190
*δα*,^f^ 100 µm (10^−4^)	150	160	77
*δα*, 1000 µm (10^−4^)	440	480	240
R_BC_,^g^ 100 µm (W/m^2^)	1.0	0.8	0.5
R_BC_, 1000 µm (W/m^2^)	3.3	2.7	1.9
R,^h^ 100 µm (W/m^2^)	2.8	1.4	0.6
R, 1000 µm (W/m^2^)	8.6	4.6	2.4

Standard deviations represent the deviations among the different sample sites. Å_tot_, ${{\rm{C}}}_{{\rm{BC}}}^{{\rm{est}}}$, ${{\rm{C}}}_{{\rm{BC}}}^{{\rm{\max }}}$, and ${{\rm{C}}}_{{\rm{BC}}}^{{\rm{equiv}}}$ represent measurements made for the surface layer; ${{\rm{C}}}_{{\rm{BC}}}^{{\rm{est}}}$, subsurf is for subsurface layers; and the final rows are for all layers. The values of albedo-reduction and radiative forcing (bottom 8 lines) are given for two snow-grain radii, 100 and 1000 µm. For 100 µm radii they are given for June at latitudes north of 33.5°S and July for the south. For 1000 µm radii they are given for July at latitudes north of 33.5°S and August for the south. ^a^Depth of snowpack. ^b^Snow water equivalent (SWE), integrated through the snowpack. ^c^Integrated black carbon (Int BC) estimate for snow pack. ^d^Average black carbon (Ave BC) estimate for snowpack, as Int BC/SWE. ^e^Albedo reduction for BC-only. ^f^Albedo reduction for all light-absorbing particles in snow. ^g^Radiative forcing, BC-only. ^h^Radiative forcing, all light-absorbing particles.

### Albedo reduction and radiative forcing

Figure 4a,b show the cloud fraction and shortwave flux for the Austral winter months June, July, and August. While cloud fraction does not change much with month, it increases substantially from north to south. Shortwave flux changes considerably with both latitude and month.

Figure 4c shows the albedo reduction due to all light-absorbing impurities in snow for each site shown in Table 5 (for the values of ${{\rm{C}}}_{{\rm{BC}}}^{{\rm{equiv}}}$ given in the table) assuming snow grain radii *r* = 100 µm (small symbols) and *r* = 1000 µm (large symbols). The choice of these representative grain sizes is explained by Wiscombe and Warren^35^ and Warren^36^. Because new snow is typically fine-grained, the albedo reduction is plotted for *r* = 100 µm for the beginning of winter (June in the north; July in the south). By contrast, melting snow is typically coarse-grained; thus the albedo reduction is plotted for *r* = 1000 µm for the melt season (July in the north, August in the south). The albedo reduction and radiative forcing are greatest in the far north and in the Santiago region.

Albedo reductions and radiative forcings for regional averages are given in Table 6 for 100 and 1000 µm snow grains and for BC-only and all light-absorbing impurities.

## Discussion

### Latitude dependence of non-BC light-absorbing impurities

**Figure 6:**
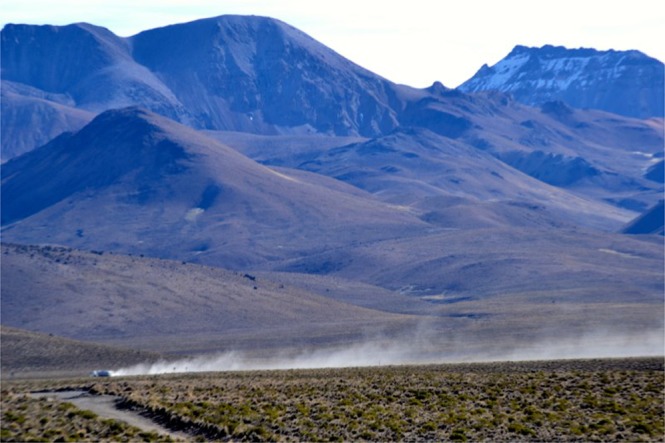
Dust trail from a truck in northern Chile. Photograph was taken by the authors.

The contribution of non-black carbon constituents to particulate absorption shows a trend with latitude, decreasing southward. The fraction of absorption from non-BC decreases from around 0.9 near San Pedro to 0.1 at the southern-most site of Osorno (Fig. 5c). The far north is a desert where large amounts of dust may be lofted into the air by natural and human causes (see, e.g. Fig. 6). In contrast, the southern region is characterized by frequent precipitation and the atmosphere is therefore less dusty.

How much the Angstrom exponent, Å_tot,_ deviates from 1.1 is an indication of the relative contribution of non-BC particles to the light-absorption. Starting at the north end of Chile and moving south, Å_tot_ first increases from about 2.5 at Putre (Site 1), to a maximum of ~5 at La Ola (Site 3; Fig. 5b), then decreases to a minimum of 1.1–1.9 at Osorno (Site 22), indicating minor contribution from non-BC at Osorno. This geographic trend in Å_tot_ holds for samples from all layers (Fig. 5b). For surface samples, the trend is also the same, except that Å_tot_ peaks at Site 2 above San Pedro (Å_tot_ = 4.5; Table 5).

Particles with Å_tot_ near 1.0 are essentially black, indicating their light-absorbing component is nearly all black carbon (Bond *et al*.^16^), indicating a combustion source for particulate matter. The low values of Å_tot_ at our southern sites are consistent with a combustion source for the snow particulate matter. Added to the knowledge that wood-burning is the main source of heating in this region and the lack of dust sources we infer that light-absorbing particles in snow are dominated by carbonaceous aerosols. To the north, the high values of Å_tot_ of 3 to >5 indicate a much larger contribution from non-BC sources. Previous measurements with the ISSW coupled with chemical analyses (refs^26,28,29^) indicate that these high values of Å_tot_ could be due to very high brown carbon to black carbon ratios in combustion-sourced aerosol or, more likely for the highest values seen here (>3), due to dust. Dust as the dominant source of light-absorbing particles to snow in northern Chile is consistent with the low population density, the proximity of dust sources and the low snow-cover fraction.

Precise attribution of absorption to sources using Å_tot_ is not possible. We have assumed that Å_NBC_ is 5.0. If the particulate absorption at Site 3 is in fact essentially all due to non-BC (e.g. dust), this supports the validity of using Å_NBC_ = 5.0 for this region. If, in fact, there is a significant contribution to absorption by BC even at Site 3, Å_NBC_ should be higher; in this case, our reported values of ${{\rm{C}}}_{{\rm{BC}}}^{{\rm{est}}}$ will be too low and, correspondingly, our attribution of absorption to brown carbon and dust will be too high. Regardless, trends in Å_tot_ are strongly indicative of compositional trends in light-absorbing particulate matter in snow.

### Latitude dependence of black carbon

As shown in Fig. 5a, ${{\rm{C}}}_{{\rm{BC}}}^{{\rm{est}}}$ decreased southward to a minimum above Elqui (Site 4), then increased continuing south to the Santiago area (Sites 7–11), and finally decreased gradually southward to Osorno (Site 22). In the north, this trend is consistent with the trend for non-BC, suggesting that the snow becomes overall cleaner moving south from the far north of Chile (Putre) to Elqui.

The trend for ${{\rm{C}}}_{{\rm{BC}}}^{{\rm{est}}}$ described above is in keeping with the regional averages, for which ${{\rm{C}}}_{{\rm{BC}}}^{{\rm{est}}}$ is highest near Santiago. Near Santiago there is a lot of variation in ${{\rm{C}}}_{{\rm{BC}}}^{{\rm{est}}}$ from site to site, which may be linked to the influence of dry deposition, as will be discussed below.

The integrated black carbon is a metric for total BC deposition over the period of snowpack accumulation. For regional averages, both integrated black carbon and snow depth increased southward. ${{\rm{C}}}_{{\rm{BC}}}^{{\rm{est}}}$ for surface samples in the Santiago region is higher than the value for southern Chile; however Santiago has considerably less integrated BC. The values for average BC are the same. The explanation is that there can be high BC concentrations but low column-integrated BC mass if the snowpack is thin.

### Wet vs. dry deposition

The above observations alone do not permit unambiguous identification of the dominant mechanism of BC deposition (dry deposition, in which BC settles onto snow from the atmosphere, or wet deposition, in which BC is transferred to snow via precipitation), because of a number of complicating factors. One complication is that the sets of numbers used to compute ${{\rm{C}}}_{{\rm{BC}}}^{{\rm{est}}}$, the integrated BC, and the subsurface BC vary slightly due to missing data and variations in snow depth (e.g. there was not always a subsurface layer to sample). This is particularly important for the Santiago area (as well as in the north), where snow depth was typically thinner and where there were large variations in ${{\rm{C}}}_{{\rm{BC}}}^{{\rm{est}}}$ (110%) and integrated BC (66%). In southern Chile the variations were smaller, 70% and 52%, respectively, indicating a more uniform snowpack. It is furthermore worth noting that if snow cover is intermittent at a given location, then the column-integrated BC mass will not reflect total BC deposition for the winter, whereas if there is no loss of snow particulate matter to melting, the column-integrated BC will reflect the total BC deposition since snowfall started. There are, however, situational factors that help constrain this distinction; an exploration of wet vs. dry deposition using a few case studies follows.

Meteorological data and observations suggest a lack of snowfall in northern and central Chile from 1 to 6 July 2015, with the exception of a light snowfall on 4 July at Site 1, Putre (observed by the sampling group). On 5 and 6 July a cold front may have resulted in some snowfall in the mountainous region east of Santiago. Another front brought snowfall to the Santiago area on 11 and 12 July and to Elqui on 13 July. Blizzard conditions with snow and wind were experienced at La Ola on 13 July, the day before sampling was performed. On 16 July a less intense front brought some snowfall to the mountains east of Site 4, Elqui, but no precipitation was measured at the station near the Elqui sampling site. There was no evidence of additional snowfall for the remainder of the 2015 field season. Further details are given in the Supplemental.

Based on the above as well as reports from locals near the field sites, Table 7 gives estimates of days since last snowfall, ${{\rm{C}}}_{{\rm{BC}}}^{{\rm{est}}}$ for both the surface and sub-surface snow, and integrated BC for many of the sites for the 2015 field season.

**Table 7:** Estimated number of days since the last snowfall before sampling, and median surface and subsurface black carbon concentration s, in northern and central Chile.

Date	Location	Days since snowfall	${{\bf{C}}}_{{\bf{BC}}}^{{\bf{est}}}$ surface (ng/g)	${{\bf{C}}}_{{\bf{BC}}}^{{\bf{est}}}$ subsurface (ng/g)
2015/07/14	La Ola	1	5	0
2015/07/17	Elqui	4	2	1
2015/07/19	Las Ramadas	6	5	—
2015/07/20	Choapa	7	9	1
2015/07/21	Portillo	8	6	7
2015/07/22	Juncal	9	14–18	6
2015/07/24	Valle Nevado	11	46–110	12
2015/07/26	Valle Maipo	13	21–31	6
2016/07/25	Yerba Loca	0	1	—
2016/07/26	Yerba Loca	1	9	—

**Figure 7:**
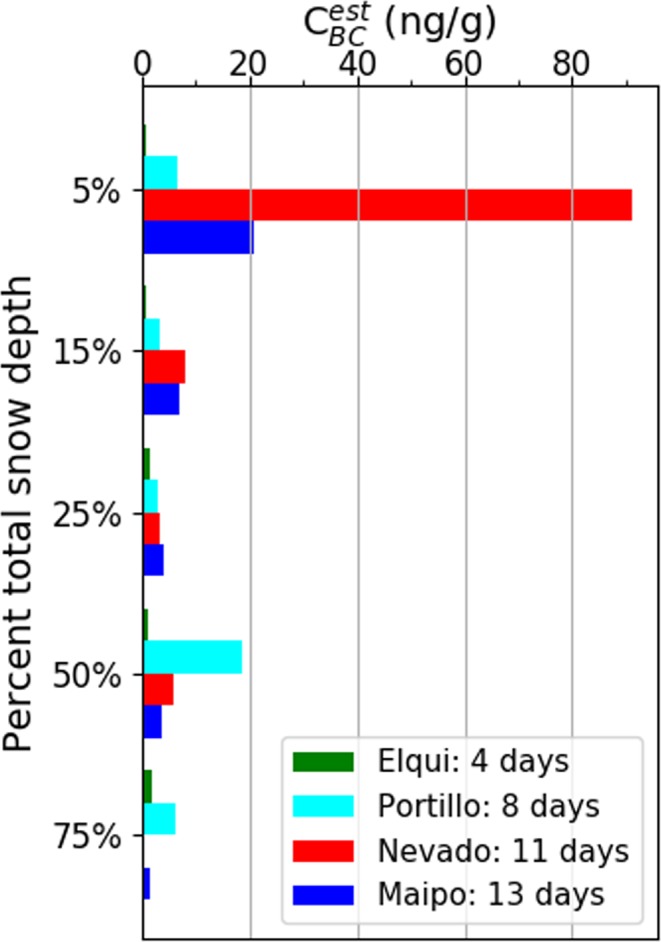
Vertical profiles of BC. The four sites all experienced snowfall in the same storm. The sampling progressed from north to south over 9 days; the estimated number of days since snow fell (4–13 days) is given for each site.

In northern Chile and the Santiago area, the date of the most recent snowfall could be estimated from observations and weather reports. As shown in Table 7 and in Fig. 7, the contamination of the surface layer and the integrated black carbon mass both generally increased with time. Given that the subsurface samples are relatively clean everywhere, proximity to Santiago seems unlikely to be a major factor. Instead, the relative dirtiness of the surface snow suggests that dry deposition plays a major role. In addition, consolidation of BC in the surface layer with snow melt^17^ may be playing a role, as well as sublimation of the surface snow at high elevations in the dry Andes^37^.

Data from meteorological stations suggest that most of the precipitation from 27.2 to 33.4°S was due to the passage of a front on 12 July, with small amounts of precipitation in this latitude range the following day for stations from 33.3 to 33.4°S. However, precipitation was measured at Santo Domingo at 33.39°S on 5 July. Thus it is possible some of the subsurface snow sampled at Valle Nevado and Valle Maipo was seven days older, consistent with the dirtier subsurface snow observed there.

Near Santiago, measurements made on sequential days more clearly indicate the importance of dry deposition. At Yerba Loca, near Santiago, the full column of the snowpack was sampled two days in a row (25 and 26 July 2016) at nearby locations, allowing for determination of the dry-deposition rate. The snow was melting and the depth decreased from 10 to 5 cm, while the snow density increased from 0.19 to 0.44 g/cm^3^. The snow-column-integrated BC increased from 31 to 200 µg/m^2^, indicating that the new snowfall in the Santiago area was relatively clean, and light-absorbing impurities arose mainly from dry deposition.

### Aerosol deposition rate near Santiago

The freshly fallen snow of 25 July 2016 at Yerba Loca was quite clean both in terms of the BC concentration and in terms of the non-BC contribution to absorption, which was negligible. On 26 July particulate absorption included a significant contribution by non-BC components, as Å_tot_ increased from ~1.1 to 2.0 and ${{\rm{f}}}_{{\rm{NBC}}}^{{\rm{est}}}$ increased from a negligible value to 0.3. Since no snow fell between these two days, this contribution must have been via dry deposition.

The deposition rate is estimated as the difference in integrated BC mass divided by the time difference, or 17.3 ng/cm^2^-day. This is equivalent to 5.2 mg/m^2^-month, which is roughly comparable to the estimated inter-monsoon dry-deposition rate of BC in the Himalayas (see Fig. 3a in Menegoz *et al*.^38^).

### Trend in BC with distance from a major highway

To determine whether significantly more BC is deposited close to major highways, measurements were made at varying distances from the Trans-Andean highway to the southeast of Juncal, in the Juncal National Park. Measurements were made 3.64 km, 1.89 km, 1.05 km, and 0.561 km from the highway (Sites 8a-d). Moving along this transect toward the highway, the surface black-carbon estimate was 15, 15, 14, and 18 ng/g. Thus black carbon from vehicles does not seem to be preferentially deposited near the road.

### Albedo reduction and radiative forcing

Albedo reduction and radiative forcing due to impurities in snow in the Chilean Andes are found to be greatest in the far north and in the Santiago region. For example, under all-sky conditions, mean albedo reductions due to light-absorbing impurities in snow in the Chilean Andes are estimated to be 0.0150, 0.0160, and 0.0077 for northern Chile (in June), the region near Santiago (in June), and southern Chile (in July), respectively, for snow grain radii of 100 µm (representative of new snow). Likewise, mean radiative forcings are estimated to be 2.8, 1.4, and 0.6 W/m^2^, respectively.

In northern Chile, BC plays a smaller role in albedo reduction than non-BC. For example, albedo reduction for 100 µm snow grains due to BC alone is only about 43% of that for all light-absorbing impurities. By comparison, these albedo reductions are 53% and 82% near Santiago and in southern Chile, where a greater share of light absorption is due to BC.

### Comparison to other measurements

The regional means and standard deviations of ${{\rm{C}}}_{{\rm{BC}}}^{{\rm{equiv}}}$ for northern Chile measured in this work (refer back to Tables 5 and 6) are higher than values for measurements made in the Cordillera Blanca in Peru^23^. At an altitude of ~4900 m, Schmitt *et al*.^23^ measured mean effective black carbon values of 10 ± 7 ng/g and 17 ± 12 ng/g. By contrast, for the three sample sites at around 5000 m for this work, the mean value of ${{\rm{C}}}_{{\rm{BC}}}^{{\rm{equiv}}}$ was 112 ± 29 ng/g. The mean for northern Chile, corresponding to altitudes of 1800–5300 m, was 72 ± 17 ng/g.

Regional averages of ${{\rm{C}}}_{{\rm{BC}}}^{{\rm{est}}}$ in the top 5 cm of snow in the Arctic and North America were 27 ng/g and 29 ng/g (ref.^33^). This is comparable to the regional average near Santiago (28 ng/g; refer to Table 6), but is about twice as large as the regional averages for northern and southern Chile (~14 ng/g). The subsurface snow was clean in the Andes (7 to 15 ng/g) compared to snow in the Arctic and North America (21 and 30 ng/g; ref.^33^).

In northern Chile, a large fraction of the computed albedo reduction is due to non-BC. When only black carbon is considered, the albedo reduction due to BC is about 43% of that for all light-absorbing impurities (Table 6). This value and the value for snow near Santiago (53%) are roughly comparable to values for the Arctic, western North America, and China (53%, 49 to 78%, and 41%). By contrast, in southern Chile, black carbon accounts for a greater share of the albedo reduction (82%).

## Conclusions

Based on snow-sampling measurements made in 2015 and 2016, the black-carbon mass-mixing ratio in snow in the Chilean Andes decreased southward from Putre (18.1°S) to the Elqui Valley above La Serena (29.9°S), after which it increased continuing southward to the Santiago area (33.3°S), then again decreased from the Santiago area south to Osorno (41.1°S).

The contribution of non-BC light-absorbing components to particulate absorption in snow decreased dramatically from north to south, as expected given the transition from the dusty, vegetation-sparse Atacama Desert to the wetter, vegetation-rich south of Chile.

Regional results for Chile are as follows: for surface snow, the average mass mixing ratio of BC was 15 ± 4 ng/g in the north, 28 ± 35 ng/g near Santiago, and 13 ± 3 ng/g in the south. The regional average vertically-integrated loading of BC was 207 ± 46 µg/m^2^ in the north, 780 ± 510 µg/m^2^ near Santiago, and 2500 ± 440 µg/m^2^ in the south, where the snow season was longer and the snow was deeper.

The estimated black carbon mass mixing ratio in surface snow generally increased with time since the last snowfall for a transect from northern to central Chile from 26.5 to 33.5°S, indicating that dry deposition plays a larger role than wet deposition. Sampling on two consecutive days near Santiago indicated a dry deposition rate of 5.2 mg/m^2^-month, which is roughly comparable to the inter-monsoon dry deposition rate in the Himalayas.

Mean albedo reductions due to light-absorbing impurities in snow in the Chilean Andes are estimated to be 0.0150, 0.0160, and 0.0077 for northern Chile (in June), the region near Santiago (in June), and southern Chile (in July), respectively, for snow grain radii of 100 µm (representative of new snow). Corresponding mean radiative forcings are 2.8, 1.4, and 0.6 W/m^2^, respectively. In the north, BC plays a smaller role than non-BC; for example, the albedo reduction for 100 µm snow grains due to BC alone is only about 43% of that for all light-absorbing impurities. By comparison, these albedo reductions are 53% and 82% near Santiago and in southern Chile, where a greater share of light absorption is due to BC.

## Supplementary information

Supplemental

## Data Availability

The datasets generated and analyzed during the current study are available from the corresponding author on reasonable request. Correspondence and requests for data should be addressed to P.M.R.
