# Supplementary material for: Black carbon and other light-absorbing impurities in snow in the Chilean Andes
Source: Sci Rep. 2019 Mar 8;9:4008. doi: 10.1038/s41598-019-39312-0 (PMC6408441; doi:10.1038/s41598-019-39312-0)
Supplement: Supplementary file 1 — Supplemental [file 41598_2019_39312_MOESM1_ESM.docx]

**Black carbon and other light-absorbing impurities in snow in the Chilean Andes**

**Penny M Rowe**^1,2^, Raul R. Cordero^1^, Stephen G. Warren^3^, Emily Stewart^4^, Sarah J. Doherty^5^, Alec Pankow^4^, Michael Schrempf^6^, Gino Casassa^7,8^, Jorge Carrasco^7^, Jaime Pizarro^1^, Shelley MacDonell^9^, Alessandro Damiani^1,10^, Fabrice Lambert^11,13^, Roberto Rondanelli^12,13^, Nicolas Huneeus^12,13^, Francisco Fernandoy^14^ and Steven Neshyba^4^

**Supplemental Information**

**S1 Site descriptions**

Sampling sites are shown on a topographic map of Chile in Figure S1. Site numbers for the Santiago region are given in an expanded view in Figure S2.

***Sites in Northern Chile.*** These sites refer to Figure 1a and Figure S1.

Site 1: Taapacá, in the Nevados de Putre, northeast of Arica (blue marker).

The snow level was above 5300 m on 2 July 2015. However, a light snowfall occurred on the evening of 3 July; the next day we sampled the thin layer of new snow on the mountain of Taapacá at 4700 m (Site 1a). This snow was therefore known to be fresh snowfall. Because the snow was too thin for us to collect surface snow without contamination by the underlying soil, we collected it instead from the surfaces of a hard plant called Llaretta, shown in Figure S3a. The following day we climbed to the nearby permanent snowfield at 5370 m (Site 1b), shown in Figure S3b. We sampled a thin upper layer of soft new snow from the recent snowfall, as well as the layer below, which was hard-packed ice-like snow, probably from melting and refreezing.

Site 2: Cerro Toco and the ALMA astronomical observatory, above San Pedro de Atacama (green marker). Site 2a. At Cerro Toco, snow was sampled on a permanent snowfield on the side of a mountain, down to a depth of 80 cm. The first 10 cm was new snow that had fallen 2 days earlier, according to the observatory team stationed at Cerro Toco. Below 10 cm the snow was crusty. Site 2b: Snow was sampled on a flat plain near the ALMA observatory; total depth was 15 cm.

Site 3: La Ola, northeast of Copiapó (yellow marker). Snow was sampled just following a snowstorm with strong wind. Sites 3a and 3b were 660 m from the road, where the snow was 13 cm deep. Site 3c was 1.6 km from the road.

Site 4: Valle del Elqui, east of La Serena (orange markers). Snow was four days old (from the same snowfall event we had experienced at La Ola), 22 cm deep, and melting. Site 4b: snow crystals were larger than at site 4a, and the snow was harder.

Site 5: Las Ramadas, in the Limarí province (red marker). The snow was thin (8 cm) and melting. According to local weather reports, the snow apparently fell in the same snowfall event as at Sites 3-4, six days prior.

Site 6: Valle Choapa, east of Salamanca (purple maker). This snow was apparently from the same snowfall event we had experienced at La Ola, and would therefore be 7 days old. Site 6a was on a dry lake bed; the snow was melting, and human footprints were visible. Site 6b was farther from the road on a sloping pasture, but no animal footprints were visible; the snow was wet.

***Sites near Santiago*.** These sites refer to Figure 2 and Figure S2.

Site 7: Portillo, northeast of Santiago (blue marker) at 2800 m. This site was on a spit of land protruding into an unfrozen lake by a major ski resort. Snow was 8 days old according to personnel at the resort. The ski season had just begun; the area had been devoid of snow until this recent snowfall, apparently the same one we had experienced at La Ola.

Site 8: Juncal, near the Trans-Andean Highway just below Portillo (black markers). Expecting to find soot-pollution from the exhaust of diesel trucks in the nearby snow, we carried out a transect up the Juncal valley, collecting samples at distances 0.6, 1.1, 1.9, and 3.6 km from the highway.

Site 9: Yerba Loca, east and slightly north of Santiago (red markers). Site 9a was located on the eastern slope of a valley, ~300 m before Los Hornitos, at 2413 m, and was visited on 18 June 2016. Site 9b was located at 1822 m, ~150 m from the hillside road, and was visited five weeks later, on 25 July 2016. Snow was falling shortly before sampling. Site 9c was at the same location as site 9b, one day later (26 July 2016); the intent was to compare the previous fresh snowfall to day-old snowfall. The snow temperature was 0 °C both days and the snow compacted from 10 cm to 5 cm.

Site 10: Valle Nevado (brown markers). Snow was sampled off the road to the ski resorts just east of Santiago, to investigate the effect of pollution coming from the large city. For Sites 10a and b sampling took place just below the Valle Nevado ski resort, 210 m from the road, ~300 m below a cliff. Black specks were visible on the snow surface. For Sites 10c and 10d sampling was near Farellones, 144 m from the road, ~100 m away from nearby houses. The snow surface was melting. Sites 10a-d were all visited on 24 July 2015. Site 10e was visited over a year later, in August 2016. At Site 10e, samples were collected at the top of a hill south of the Valle Nevado ski resort. The snow surface was smooth and dense (feet did not sink completely). Snow was homogeneous with depth, with no changes in texture observed.

Site 11. Valle Maipo, east of Santiago (green markers). For sites 11a-c, sampling was done in Valle Maipo and Rio Colorado at ~2300 m. The weather was warm and cloudy, and the snow was melting. Sites 11a and b were near each other; 11c was a short distance down the road (the green markers for 11a-c overlap in Figure 2). At site 11d, snow was sampled the following day off the Termas road at the southeast end of the Maipo valley at 2392m (this site is just off the map in Figure 2, and is indicated by the second green marker in Figure 1b). There was traffic on the nearby dirt road.

***Sites in Southern Chile*.** Sites refer to Figure 1b and Figure S1.

Site 12: Curicó (blue marker south of Rancagua). Sampling was done on the ledge of a hill. The snow surface was smooth, fairly homogeneous, and dense.

Site 13: Laguna del Maule (green marker north of Chillan): The sampling area was on the slope of a hill northeast of Lake Maule, ~200 m from the main road. The snow surface was a breakable crust over low-density snow. Within the snow column were three distinct snow textures: 0-35 cm was irregular particles and/or crowned columns, 35-80 cm was larger pellet crystals that could be differentiated with the naked eye, and 80-100 cm was smaller pellet crystals.

Site 14: Las Trancas, near Termas de Chillan (yellow markers). Site 14a, sampled on 13 July 2016, was in a clearing on a slope, surrounded by trees. Site 14b was sampled 6 weeks later, on 22 August 2016, in a valley next to a slope of the volcano southeast of the Nevados de Chillan. A river was ~20 m from the site, and trees ~10 m away. The snow surface was rough, with concave grooves, but no apparent color. The surface was brittle (poor load-bearing capacity). Below 15 cm the grains became larger and more translucent.

Site 15: Antuco (orange marker). The samples were taken in a valley northeast of the Antuco Volcano, between the volcano and the Velluda mountain range. The surface was rough, with convex grooves, and brittle.

Site 16: Volcan Collaqui (red marker). The sampling area was located in a valley by the road that ascends the Collaqui volcano.

Site 17: Corralco (purple marker). Samples were collected on a low slope (~10°) northwest of the Corralco Ski Center, ~3.6 km from Lonquimay Volcano. Snow depth was 80 cm, with varying texture, hardness and impurities. In the first 10 cm, the snow appeared clean, but not fresh, since it compacted only ~3 cm when walking on it. Between 10 and 40 cm, the snow was harder, with coarser grains and isolated visible impurities. Between 40 and 45 cm was a layer of dark snow, with a high concentration of impurities; snow grain size was similar to the layer above. Between 50 and 60 cm a second layer of accumulated impurities was visually evident, separated by ~10 cm from the previous one. At the bottom of the snow column, the grain size was larger.

Site 18: Lonquimay (black marker). The site was at the top of a small hill, ~1000 m southwest of the Arenales Ski Center, which was inactive during sampling, and ~150 m from the R-89 road. The surface snow was smooth and brittle. The first 10 cm was snow that had fallen the previous night. Between 15 and 45 cm were pellets of smaller size and from 45 to 60 cm were larger pellets.

Site 19: Volcan Llaima (green marker). The snow, collected on a slight slope, was stratified, with a very thin surface layer (~1 cm) of compacted, coarse-grained snow. Immediately below was ~4 cm of soft, fine-grained snow, probably fairly fresh. The snow below was harder and coarse-grained.

Site 20 Villarica (yellow marker): Site 20a was at a resort on Volcan Villarica outside the Pucon area, close to a ski lift at ~1357 m. The snow was old, icy, 65 cm deep. Eight samples were collected down to 35 cm. Site 20b was located near a canyon oriented approximately North-South, a few meters above the vegetation line at the southern hillside of the volcano. The sample site was away from the ski track, immediately south of the ski lifts, where the slope was slight and the snow was compacted.

Site 21: Antillanca ski resort in Parque Nacional Puyehue (orange marker). Site 21a: ~1093 m. Samples were collected on the side of a ski slope facing south up the mountain. There were animal tracks and footprints nearby. The snow was 26 cm deep, but samples were collected only down to 10 cm. Site 21b: Samples were collected from the west sector of the Antillanca mountain range, ~400 m from the road near the Rayhuen crater. To the west Puyehue Lake was visible, and to the south the Puntiagudo Volcano was visible. The snow was very dense in the first 10 cm, almost ice, making the extraction process difficult. Below this layer the snow was less compacted; from 10 to 15 cm, the snow was soft and fresh, from the recent precipitation event (1 or 2 days ago).

Site 22: Volcan Osorno in Parque Nacional Puyehue (red marker). The site was on the west side of the volcano, ~500 m northeast of the ski center, on a 20-30° slope, within sight of Llanquihue Lake and Calbuco Volcano to the west. Total depth was typically about 40 cm, but the snow was deeper at the sampling point (~70 cm). Stratification was apparent, with a 10-cm-thick surface layer of thin, fine-grained snow. Impurities were visible in the bottom 5 cm of this layer. Below this, the snow was thicker and compacted, probably from to an older deposition event.

**S2 Measurements of light-absorbing impurities in snow**

Tables S1-S3 give the median values for measurements of for $Å_{\mathrm{tot}}$,$C_{\mathrm{BC}}^{\mathrm{est}}$ , $C_{\mathrm{BC}}^{\max}$, and $C_{\mathrm{BC}}^{\mathrm{equiv}}$ at each location for each layer of snow. Medians were calculated for multiple samples made within the same layer (typically two measurements were made within each layer) at a given site.

**S3 Visual estimates of BC equivalent loading**

In our field laboratories, as soon as the filters are dry they are compared visually to a set of calibration standards containing known loadings of Fullerene, so as to help in planning for our subsequent days’ sampling, as was done on previous expeditions (see e.g. ref 27). In this work, comparison is done in two ways: (1) with filters held against a white background (the light reflection method), and (2) by viewing artificial light through the filter, by placing measured and standard filters on a light table (the light transmission method). While the light reflection method was used in prior work, the transmission method is new to this work. Figure S4a shows the error in our visual estimates, by reference to the measured BC equivalent determined using the ISSW. The reflection method typically underestimates the BC-equivalent loading by about 50% (blue points in S2b). This is in agreement with some previous work,^29^ while other work found a smaller bias.^26^ (Note that we plot the equivalent BC loading in μg/cm^2^ rather than converting to ng/g as in previous work, and we restrict our values to <11 μg/cm^2^, above which the filters are so dark that loadings cannot be distinguished visually). Figure S4 shows that visual estimates using transmitted light have a smaller bias than using reflected light.

**S4. Meteorology of Northern Chile**

From 1 to 6 July 2015 no distinctive pressure systems were present in northern and central Chile, with sea-level pressure varying between 1010 and 1020 hPa. (Note, however, that a light snowfall was observed by the sampling group at Site 1, Putre, on 4 July). On 5 and 6 July a cold front from the Pacific crossed central Chile, which brought rainfall to the region west and south of Santiago (e.g. 18.4 mm for Santo Domingo, 2.4 mm for Santiago, based on meteorological station data; http://explorador.cr2.cl) and this may have resulted in some snowfall in the mountainous region east of Santiago so that the 0°C isotherm was around 2500 m asl at 12 UTC on 5 July (local mountain weather stations registered 1.0 - 5.5 mm).

Following this, more rain and snow fell in central Chile due to a more distinctive front that was part of a low-pressure system, which moved from southwest of the Pacific coast towards the northeast and passed Santiago around noon on 12 July. Passage of the front is shown in Figures S5-S6. In the Santiago area and the regions south and west of it, most of the rain fell between 11 and 12 July. On 13 July the front brought rainfall to the region north of Santiago (e.g. Elqui). The front passed through Site 3, La Ola, on 13 July, bringing blizzard conditions with snow and wind the day before sampling was performed, as well as bringing snowfall to the mountainous regions in the east to the north of Santiago. On 16 July a second less intense front brought some snowfall in the mountains east of Site 4, Elqui. There were no further distinctive pressure systems and no additional rain or snowfall for the remainder of the 2015 field season.

Table S1. Median values for each layer of snow at each location in northern Chile.

| Site | Dates | Location | Latitude  (^o^S) | Elevation (m) | Sample Depth (cm) | $Å_{\mathrm{tot}}$ | $C_{\mathrm{BC}}^{\mathrm{est}}$ (ng/g) | $C_{\mathrm{BC}}^{\max}$ (ng/g) | $C_{\mathrm{BC}}^{\mathrm{equiv}}$ (ng/g) |
| --- | --- | --- | --- | --- | --- | --- | --- | --- | --- |
| 1a | 2015/07/04 | Putre | 18.1 | 4707 | 0-1 | 2.8 | 25 | 34 | 50 |
| 1b | 2015/07/05 | Putre | 18.1 | 5318 | 0-5 | 2.3 | 74 | 93 | 121 |
|  |  |  |  |  | 5-15 | 2.3 | 48 | 59 | 72 |
| 2a | 2015/07/10 | San Pedro | 23.0 | 5370 | 0-5 | 4.3 | 19 | 64 | 123 |
|  |  |  |  |  | 5-10 | 4.7 | 13 | 83 | 168 |
|  |  |  |  |  | 10-15 | 5.3 | 6 | 225 | 313 |
|  |  |  |  |  | 15-20 | 4.4 | 48 | 183 | 404 |
|  |  |  |  |  | 20-30 | 3.5 | 32 | 55 | 95 |
|  |  |  |  |  | 30-40 | 2.7 | 17 | 23 | 31 |
|  |  |  |  |  | 40-50 | 2.6 | 22 | 29 | 39 |
|  |  |  |  |  | 50-60 | 2.8 | 20 | 26 | 37 |
| 2b | 2015/07/10 | San Pedro | 23.0 | 5062 | 0-5 | 4.8 | 12 | 98 | 210 |
| 3a | 2015/07/14 | La Ola | 26.5 | 3578 | 0-13 | - | 5 | - | - |
| 3b | 2015/07/14 | La Ola | 26.5 | 3578 | 0-7 | 4.1 | 5 | 12 | 20 |
| 3c | 2015/07/14 | La Ola | 26.5 | 3624 | 0-5 | 4.1 | 5 | 12 | 25 |
|  |  |  |  |  | 5-10 | 5.7 | - | 40 | - |
|  |  |  |  |  | 10-15 | 5.6 | - | 44 | - |
|  |  |  |  |  | 15-20 | 5.2 | - | 40 | - |
|  |  |  |  |  | 20-25 | 5.0 | - | 40 | - |
| 4a | 2015/07/17 | Elqui | 29.9 | 2341 | 0-5 | 4.6 | 1 | 5 | 12 |
|  |  |  |  |  | 5-10 | 3.3 | 1 | 2 | 4 |
|  |  |  |  |  | 10-15 | 3.1 | 1 | 2 | 2 |
|  |  |  |  |  | 15-20 | 3.2 | 2 | 3 | 5 |
| 4b | 2015/07/17 | Elqui | 30.0 | 2190 | 0-5 | 3.5 | 2 | 4 | 7 |
|  |  |  |  |  | 5-10 | 4.0 | 1 | 3 | 5 |
| 5 | 2015/07/19 | Las Ramadas | 31.0 | 1757 | 0-4 | 3.5 | 5 | 8 | 15 |
| 6a | 2015/07/20 | Choapa | 32.1 | 1919 | 0-3 | 3.1 | 10 | 16 | 26 |
| 6b | 2015/07/20 | Choapa | 32.1 | 1902 | 0-5 | 2.5 | 7 | 10 | 13 |
|  |  |  |  |  | 4-9 | 2.9 | 1 | 2 | 3 |

Table S2. Median values for each layer of snow at each location in the Santiago area.

| Site | Dates | Location | Latitude  (^o^S) | Elevation (m) | Sample Depth (cm) | $Å_{\mathrm{tot}}$ | $C_{\mathrm{BC}}^{\mathrm{est}}$ (ng/g) | $C_{\mathrm{BC}}^{\max}$ (ng/g) | $C_{\mathrm{BC}}^{\mathrm{equiv}}$ (ng/g) |
| --- | --- | --- | --- | --- | --- | --- | --- | --- | --- |
| 7 | 2015/07/21 | Portillo | 32.8 | 2800 | 0-5 | 2.6 | 6 | 8 | 11 |
|  |  |  |  |  | 5-10 | 3.1 | 3 | 5 | 7 |
|  |  |  |  |  | 10-15 | 3.2 | 5 | 8 | 13 |
|  |  |  |  |  | 15-20 | 3.3 | 3 | 5 | 8 |
|  |  |  |  |  | 20-30 | 3.3 | 6 | 9 | 15 |
|  |  |  |  |  | 30-40 | 3.1 | 18 | 27 | 43 |
|  |  |  |  |  | 40-50 | 2.5 | 6 | 8 | 11 |
| 8a | 2015/07/22 | Juncal | 32.9 | 2249 | 0-5 | 2.1 | 18 | 21 | 25 |
| 8b | 2015/07/22 | Juncal | 32.9 | 2285 | 0-5 | 2.1 | 14 | 16 | 19 |
|  |  |  |  |  | 5-10 | 3.0 | 5 | 7 | 11 |
| 8c | 2015/07/22 | Juncal | 32.9 | 2277 | 0-5 | 2.6 | 15 | 20 | 27 |
|  |  |  |  |  | 5-10 | 3.2 | 10 | 14 | 21 |
| 8d | 2015/07/22 | Juncal | 32.9 | 2322 | 0-5 | 2.7 | 15 | 21 | 29 |
|  |  |  |  |  | 5-10 | 4.3 | 3 | 11 | 24 |
|  |  |  |  |  | 10-15 | 3.8 | 4 | 8 | 16 |
| 9a | 2016/06/18 | Yerba Loca | 33.3 | 2413 | 0-8 | 2.0 | 15 | 17 | 21 |
|  |  |  |  |  | 9-16 | 1.1 | 4 | 4 | 3 |
|  |  |  |  |  | 17-24 | 1.2 | 7 | 7 | 8 |
|  |  |  |  |  | 24-32 | 1.0 | 6 | 6 | 6 |
| 9b | 2016/07/25 | Yerba Loca | 33.3 | 1822 | 0-5 | 1.0 | 1 | 1 | 1 |
|  |  |  |  |  | 5-7 | 1.1 | 2 | 2 | 2 |
| 9c | 2016/07/26 | Yerba Loca | 33.3 | 1822 | 0-5 | 2.0 | 9 | 10 | 13 |
| 10a | 2015/07/24 | Valle Nevado | 33.4 | 2435 | 5-10 | 2.5 | 5 | 7 | 9 |
| 10b | 2015/07/24 | Valle Nevado | 33.4 | 2636 | 0-5 | 3.1 | 91 | 153 | 227 |
|  |  |  |  |  | 5-10 | 2.4 | 8 | 11 | 14 |
|  |  |  |  |  | 10-20 | 2.4 | 3 | 4 | 6 |
|  |  |  |  |  | 20-30 | 2.8 | 6 | 8 | 12 |
| 10c | 2015/07/24 | Valle Nevado | 33.4 | 2366 | 0-10 | 3.4 | 66 | 130 | 204 |
|  |  |  |  |  | 10-20 | 2.5 | 12 | 16 | 20 |
|  |  |  |  |  | 20-30 | 2.5 | 4 | 5 | 7 |
| 10d | 2015/07/24 | Valle Nevado | 33.4 | 2802 | 0-5 | 3.7 | 112 | 257 | 467 |
|  |  |  |  |  | 5-10 | 3.3 | 27 | 47 | 77 |
| 10e | 2015/07/24 | Valle Nevado | 33.4 | 2554 | 0-10 | 3.3 | 46 | 93 | 144 |
|  |  |  |  |  | 10-20 | 2.4 | 3 | 3 | 4 |
|  |  |  |  |  | 20-30 | 2.4 | 4 | 5 | 6 |
| 10f | 2016/08/18 | Valle Nevado | 33.4 | 2635 | 5-15 | 2.3 | 28 | 32 | 43 |
|  |  |  |  |  | 15-25 | 2.6 | 31 | 38 | 56 |
|  |  |  |  |  | 25-35 | 1.8 | 7 | 8 | 9 |
| 11a | 2015/07/26 | Maipo | 33.5 | 2302 | 0-5 | 3.8 | 31 | 81 | 140 |
|  |  |  |  |  | 5-10 | 2.9 | 4 | 5 | 8 |
|  |  |  |  |  | 10-15 | 3.4 | 3 | 7 | 14 |
|  |  |  |  |  | 15-20 | 3.0 | 9 | 13 | 20 |
| 11b | 2015/07/26 | Maipo | 33.5 | 2308 | 5-10 | 2.8 | 9 | 12 | 18 |
|  |  |  |  |  | 10-15 | 2.9 | 8 | 11 | 16 |
| 11c | 2015/07/26 | Maipo | 33.5 | 2236 | 0-5 | 3.5 | 29 | 62 | 103 |
|  |  |  |  |  | 5-10 | 2.9 | 7 | 10 | 15 |
| 11d | 2015/07/27 | Maipo | 33.8 | 2392 | 0-5 | 3.1 | 21 | 32 | 50 |
|  |  |  |  |  | 5-10 | 2.6 | 7 | 9 | 13 |
|  |  |  |  |  | 10-15 | 2.8 | 6 | 8 | 12 |
|  |  |  |  |  | 15-20 | 2.7 | 4 | 5 | 7 |
|  |  |  |  |  | 20-30 | 2.8 | 5 | 7 | 10 |
|  |  |  |  |  | 30-40 | 2.6 | 4 | 5 | 6 |
|  |  |  |  |  | 40-50 | 2.6 | 2 | 2 | 3 |

Table S3. Median values for each layer of snow at each location in southern Chile.

| Site | Dates | Location | Latitude  (^o^S) | Elevation (m) | Sample Depth (cm) | $Å_{\mathrm{tot}}$ | $C_{\mathrm{BC}}^{\mathrm{est}}$ (ng/g) | $C_{\mathrm{BC}}^{\max}$ (ng/g) | $C_{\mathrm{BC}}^{\mathrm{equiv}}$ (ng/g) |
| --- | --- | --- | --- | --- | --- | --- | --- | --- | --- |
| 12 | 2016/08/19 | Curicó | 35.1 | 1860 | 0-10 | 2.8 | 27 | 31 | 50 |
|  |  |  |  |  | 10-20 | 1.8 | 13 | 13 | 16 |
|  |  |  |  |  | 20-30 | 1.8 | 18 | 18 | 22 |
|  |  |  |  |  | 30-40 | 1.9 | 19 | 20 | 25 |
|  |  |  |  |  | 40-50 | 2.0 | 10 | 11 | 14 |
|  |  |  |  |  | 50-60 | 2.4 | 7 | 7 | 10 |
| 13 | 2016/08/20 | Maule | 36.0 | 1860 | 0-10 | 2.1 | 17 | 19 | 26 |
|  |  |  |  |  | 10-20 | 1.8 | 12 | 12 | 15 |
|  |  |  |  |  | 20-30 | 2.7 | 24 | 33 | 54 |
|  |  |  |  |  | 30-40 | 1.7 | 14 | 15 | 18 |
|  |  |  |  |  | 40-50 | 1.6 | 10 | 11 | 12 |
|  |  |  |  |  | 50-60 | 1.4 | 7 | 7 | 7 |
|  |  |  |  |  | 60-70 | 1.4 | 8 | 8 | 8 |
|  |  |  |  |  | 70-80 | 1.7 | 7 | 7 | 8 |
|  |  |  |  |  | 80-90 | 1.7 | 8 | 8 | 9 |
| 14a | 2016/08/22 | Chillán | 37.0 | 1963 | 0-10 | 1.7 | 15 | 16 | 20 |
|  |  |  |  |  | 10-20 | 2.1 | 8 | 9 | 13 |
|  |  |  |  |  | 30-40 | 2.3 | 10 | 14 | 20 |
| 14b | 2016/07/13 | Chillán | 37.0 | 1554 | 0-5 | 1.5 | 13 | 6 | 6 |
|  |  |  |  |  | 5-10 | 1.4 | 5 | 6 | 6 |
|  |  |  |  |  | 10-15 | 1.3 | 6 | 8 | 8 |
|  |  |  |  |  | 15-20 | 1.4 | 7 | 7 | 7 |
| 15 | 2016/08/23 | Antuco | 37.4 | 1494 | 0-5 | 1.8 | 12 | 13 | 15 |
|  |  |  |  |  | 5-10 | 2.1 | 15 | 16 | 22 |
|  |  |  |  |  | 10-15 | 2.0 | 15 | 16 | 21 |
|  |  |  |  |  | 15-20 | 1.6 | 7 | 7 | 8 |
| 16 | 2016/08/24 | Collaqui | 37.9 | 1142 | 0-5 | 1.6 | 5 | 6 | 6 |
|  |  |  |  |  | 5-10 | 1.8 | 8 | 9 | 11 |
| 17 | 2016/08/27 | Corralco | 38.4 | 1601 | 0-10 | 1.5 | 5 | 5 | 5 |
|  |  |  |  |  | 10-20 | 2.0 | 7 | 8 | 10 |
|  |  |  |  |  | 20-30 | 1.8 | 10 | 10 | 12 |
|  |  |  |  |  | 30-40 | 2.1 | 7 | 8 | 10 |
|  |  |  |  |  | 40-50 | 3.2 | 20 | 27 | 50 |
|  |  |  |  |  | 50-60 | 2.8 | 17 | 21 | 36 |
|  |  |  |  |  | 60-70 | 2.1 | 9 | 9 | 12 |
|  |  |  |  |  | 70-80 | 2.4 | 8 | 8 | 12 |
| 18 | 2016/08/26 | Lonquimay | 38.4 | 1652 | 0-5 | 1.5 | 6 | 6 | 6 |
|  |  |  |  |  | 5-10 | 2.3 | 12 | 14 | 19 |
|  |  |  |  |  | 10-20 | 2.1 | 12 | 13 | 17 |
|  |  |  |  |  | 20-30 | 2.2 | 10 | 11 | 15 |
|  |  |  |  |  | 30-40 | 2.3 | 11 | 12 | 17 |
|  |  |  |  |  | 40-50 | 1.9 | 7 | 7 | 9 |
| 19 | 2016/08/28 | Llaima | 38.5 | 1830 | 0-5 | 2.1 | 7 | 9 | 12 |
|  |  |  |  |  | 5-10 | 2.4 | 11 | 14 | 20 |
|  |  |  |  |  | 10-10 | 1.4 | 12 | 12 | 13 |
|  |  |  |  |  | 20-30 | 1.3 | 7 | 7 | 7 |
|  |  |  |  |  | 30-40 | 1.3 | 7 | 7 | 7 |

Table S3 (continued). Median values for each layer of snow at each location in southern Chile.

| Site | Dates | Location | Latitude  (^o^S) | Elevation (m) | Sample Depth (cm) | $Å_{\mathrm{tot}}$ | $C_{\mathrm{BC}}^{\mathrm{est}}$ (ng/g) | $C_{\mathrm{BC}}^{\max}$ (ng/g) | $C_{\mathrm{BC}}^{\mathrm{equiv}}$ (ng/g) |
| --- | --- | --- | --- | --- | --- | --- | --- | --- | --- |
| 20a | 2016/07/21 | Villarrica | 39.4 | 1357 | 0-5 | 1.7 | 10 | 10 | 12 |
|  |  |  |  |  | 5-15 | 1.6 | 7 | 7 | 8 |
|  |  |  |  |  | 15-25 | 1.3 | 5 | 5 | 6 |
|  |  |  |  |  | 25-35 | 1.5 | 7 | 7 | 8 |
| 20b | 2016/08/29 | Villarrica | 39.4 | 1450 | 0-5 | 2.0 | 11 | 13 | 17 |
|  |  |  |  |  | 5-10 | 1.7 | 17 | 18 | 22 |
|  |  |  |  |  | 10-20 | 1.2 | 12 | 12 | 12 |
|  |  |  |  |  | 20-30 | 1.3 | 8 | 8 | 8 |
|  |  |  |  |  | 30-40 | 1.5 | 16 | 16 | 18 |
|  |  |  |  |  | 40-50 | 1.5 | 8 | 8 | 9 |
|  |  |  |  |  | 50-60 | 1.3 | 10 | 10 | 11 |
|  |  |  |  |  | 60-70 | 1.3 | 15 | 15 | 16 |
|  |  |  |  |  | 70-80 | 1.4 | 16 | 16 | 18 |
|  |  |  |  |  | 80-90 | 1.3 | 13 | 13 | 13 |
| 21a | 2016/07/19 | Antillanca | 40.8 | 1093 | 0-5 | 1.5 | 11 | 12 | 12 |
|  |  |  |  |  | 5-10 | 1.2 | 10 | 10 | 10 |
| 21b | 2016/09/01 | Antillanca | 40.8 | 1349 | 0-5 | 1.1 | 7 | 7 | 7 |
|  |  |  |  |  | 5-10 | 1.3 | 6 | 6 | 6 |
|  |  |  |  |  | 10-20 | 1.4 | 11 | 11 | 12 |
|  |  |  |  |  | 20-30 | 1.2 | 7 | 7 | 7 |
|  |  |  |  |  | 30-40 | 1.2 | 7 | 7 | 7 |
|  |  |  |  |  | 40-50 | 1.2 | 8 | 8 | 8 |
|  |  |  |  |  | 50-60 | 1.4 | 9 | 9 | 10 |
| 22 | 2016/08/31 | Osorno | 41.1 | 1326 | 0-5 | 1.2 | 5 | 5 | 5 |
|  |  |  |  |  | 5-10 | 1.1 | 5 | 5 | 4 |
|  |  |  |  |  | 10-20 | 1.9 | 14 | 14 | 18 |
|  |  |  |  |  | 20-30 | 1.5 | 5 | 5 | 5 |
|  |  |  |  |  | 30-40 | 1.3 | 4 | 4 | 4 |
|  |  |  |  |  | 40-50 | 1.3 | 6 | 6 | 6 |

**Supplemental Figures**


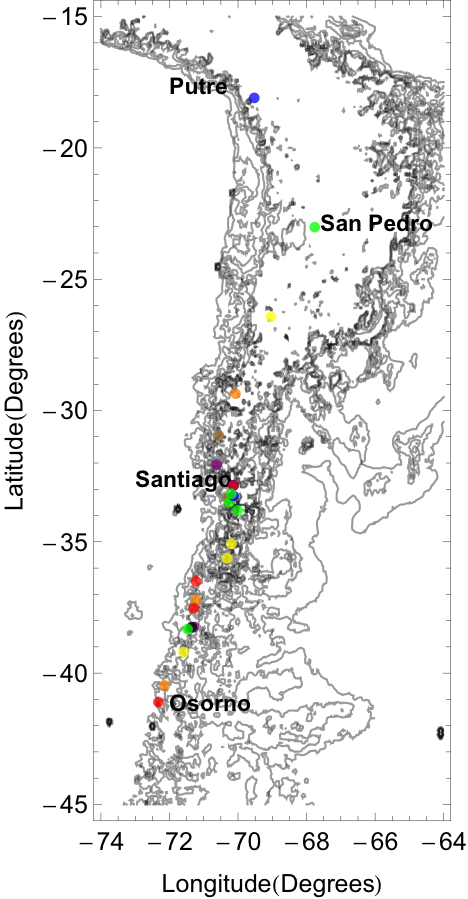


Figure S1. Sample sites.

a) b)

Figure S2. a) Sample sites in the region near Santiago. b) Elevation along latitude 33°27’S.


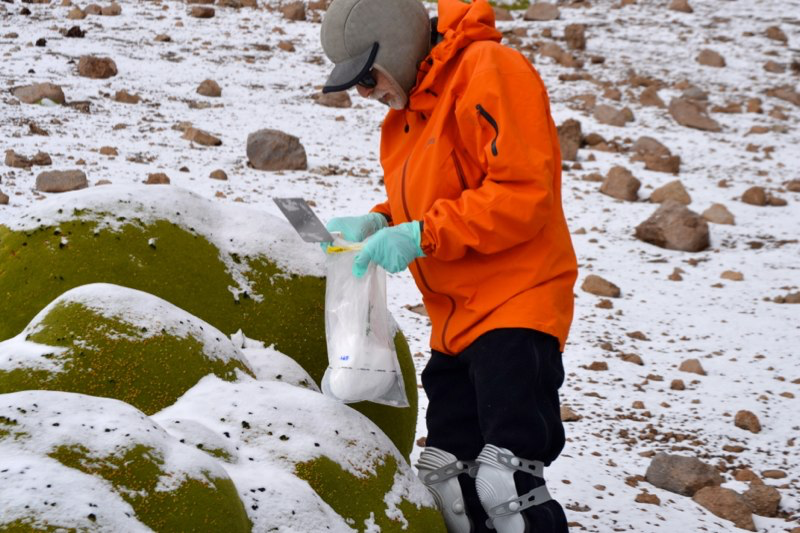


**a. Putre, site 1a.**


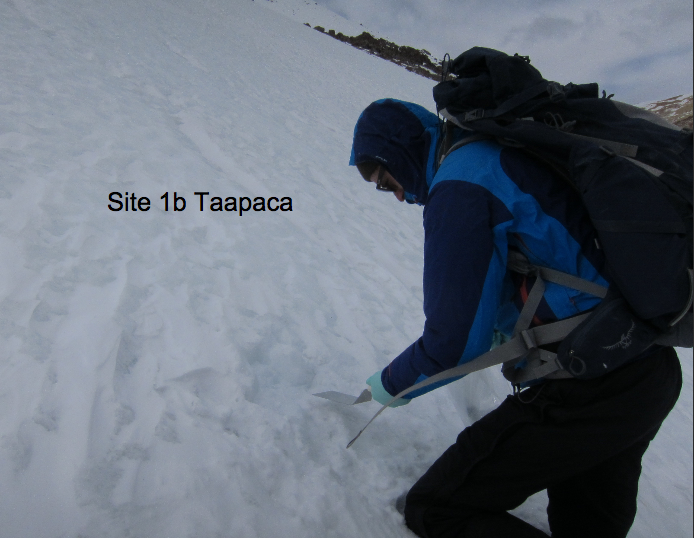


**b. Putre, site 1b.**

**Figure S3.** Snow sampling sites at Putre for (a) Site 1a, and (b) Site 1b.

**Figure S4.** (a) Visual estimates compared to measured BC equivalent. (b) Bias in visual estimate.


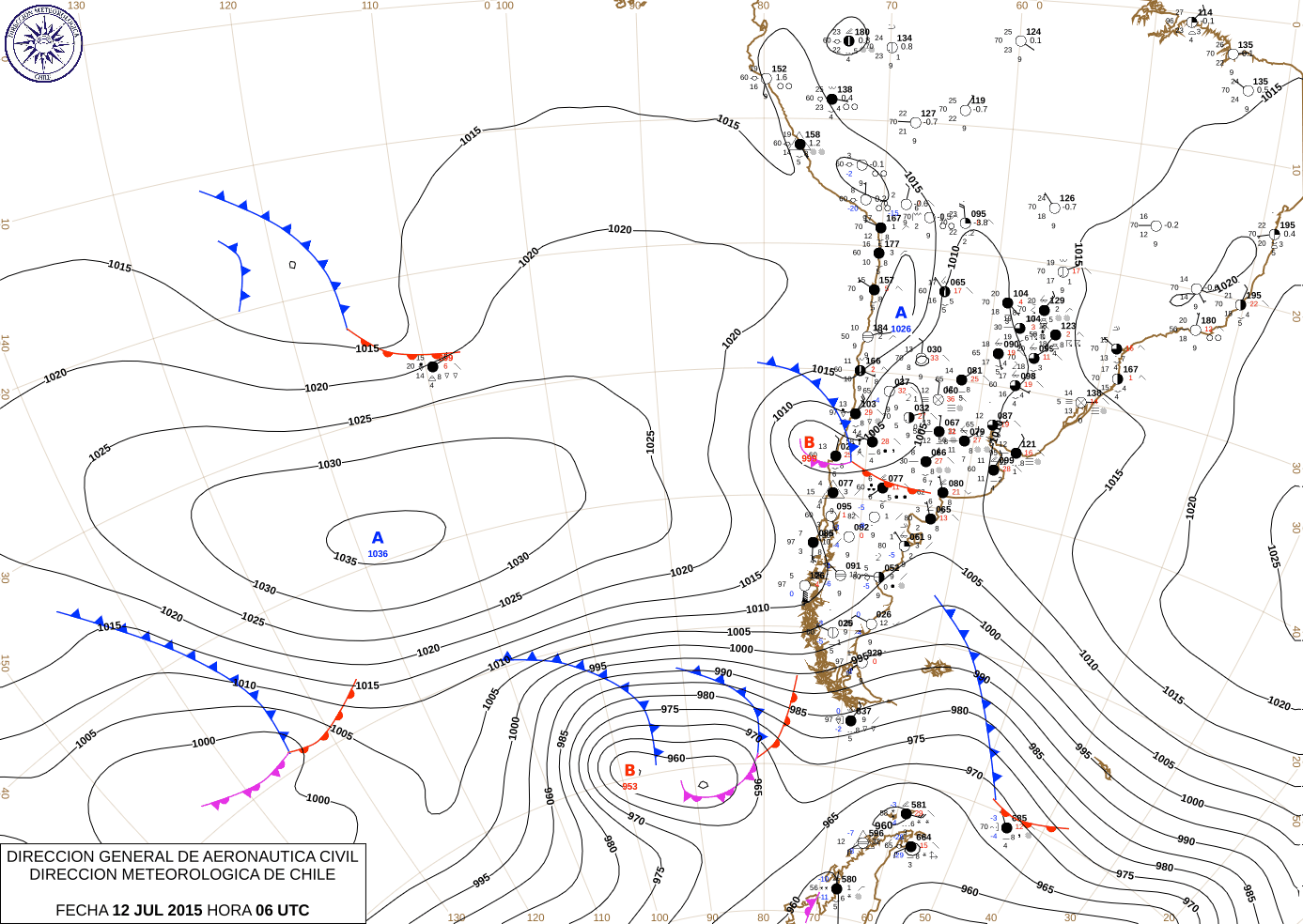


a) 12 July 06 UTC

b) 12 July 12 UTC

**
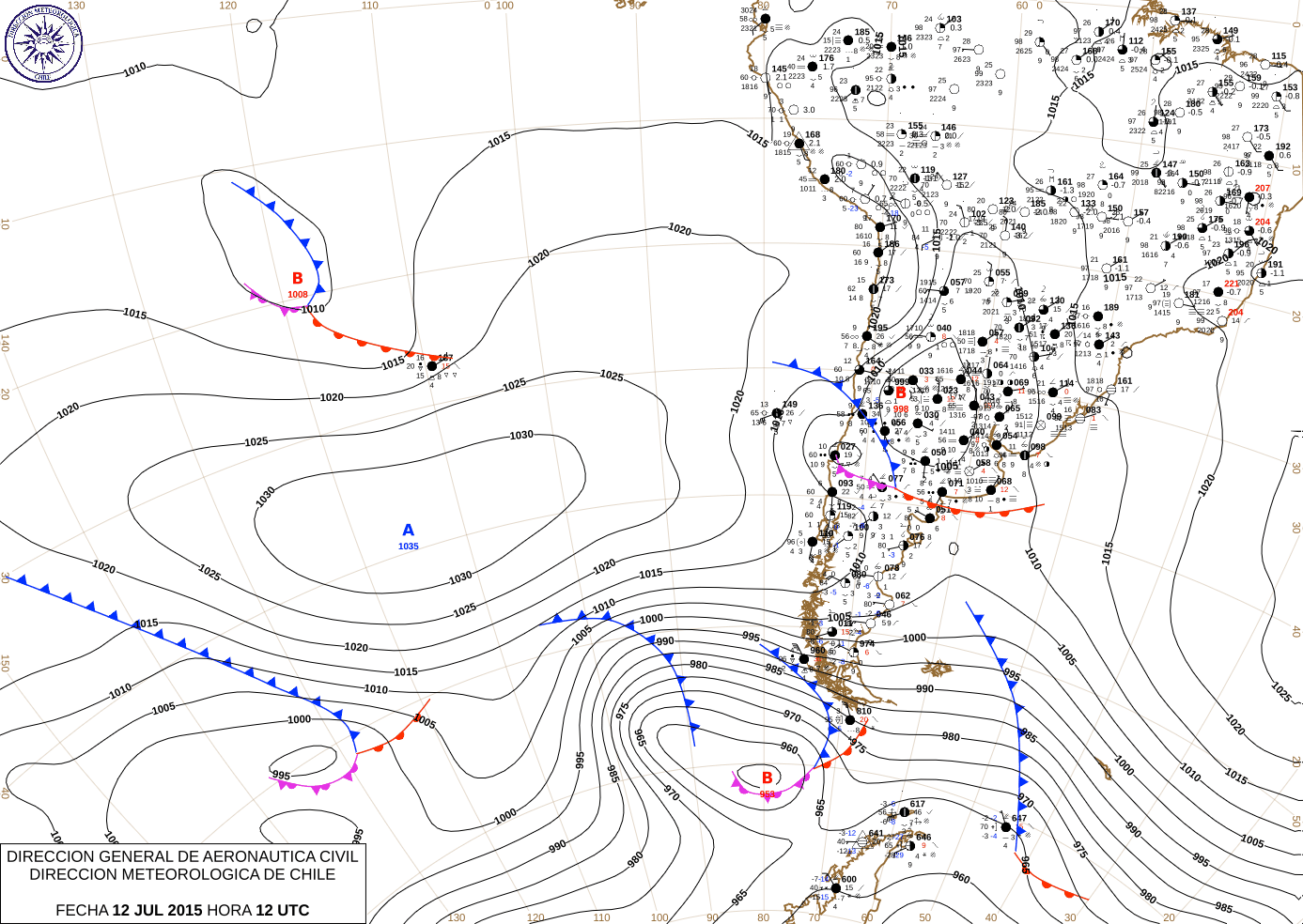
Figure S5.** Maps of the cold front on 12 July 2015 from the weather service of Chile (Dirección Meteorológica de Chile). The blue “A” stands for Alta (high), indicating a high-pressure system, while the red “B” stands for Baja (low), indicating a low-pressure system.


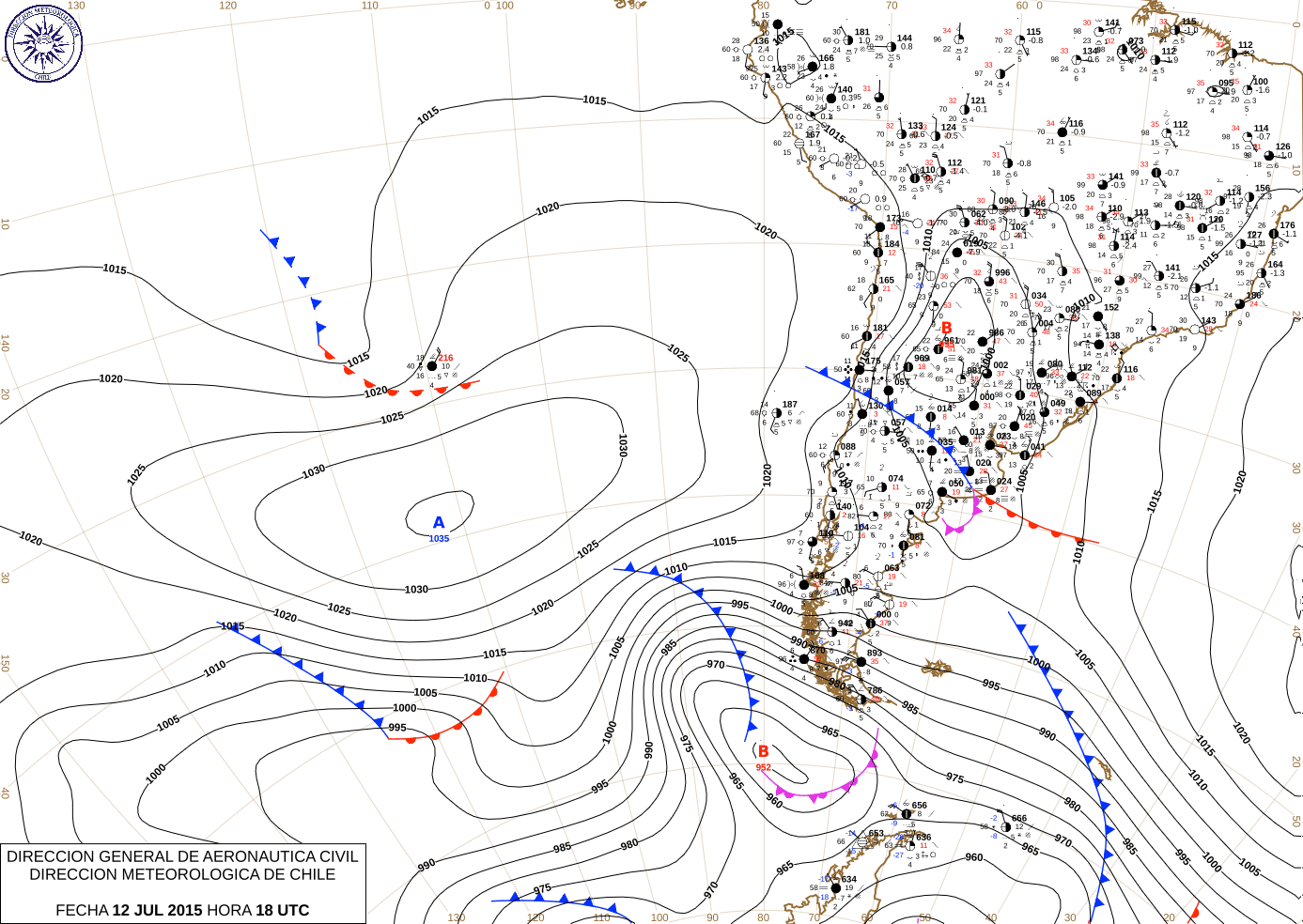


b) 13 July 06 UTC

a) 12 July 18 UTC

**
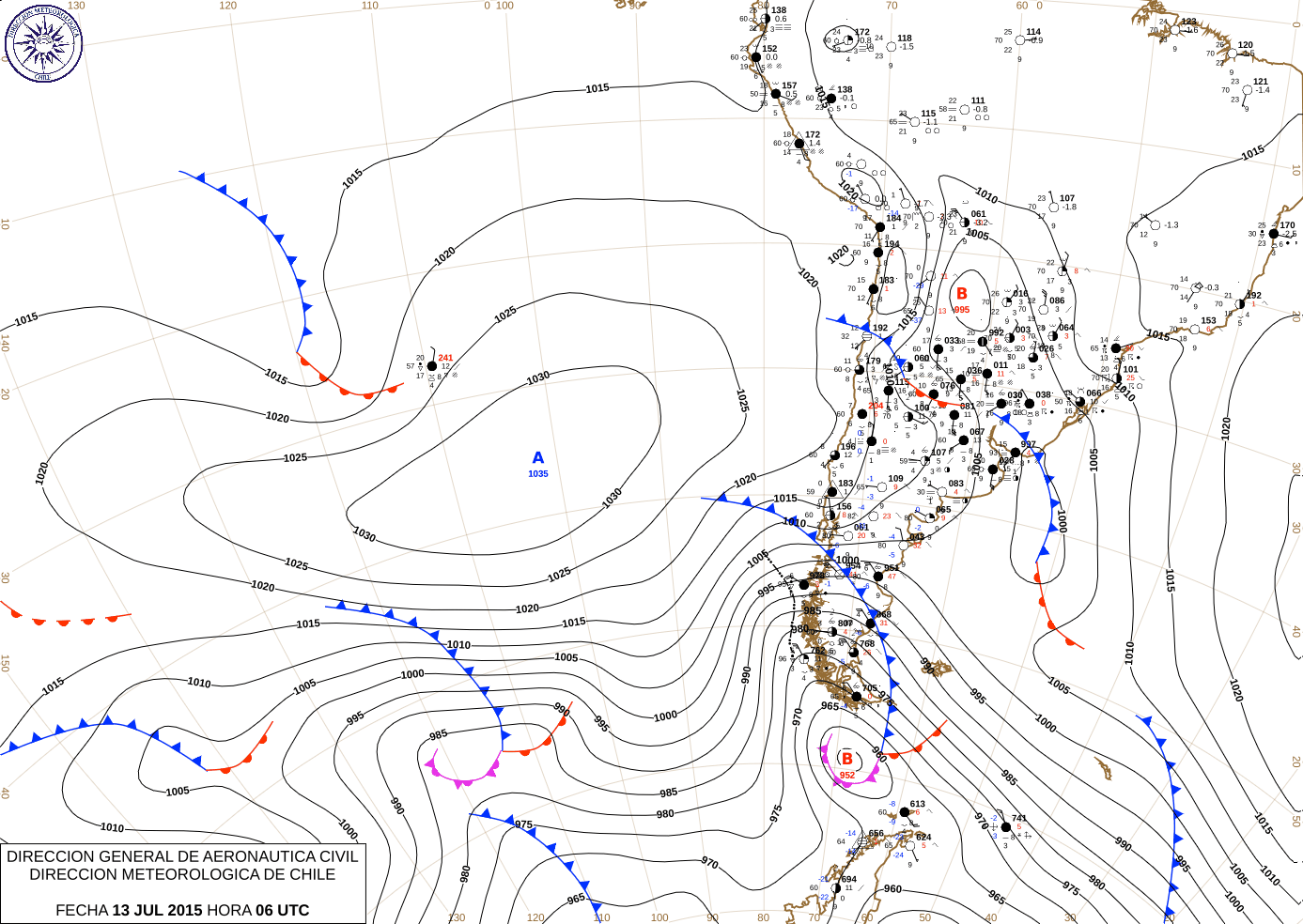
Figure S6**. Maps of the cold front on 12 July 2015 from the weather service of Chile (Dirección Meteorológica de Chile).
